# Simulation Bridges LGBTQ+ Educational Gaps in Gynecologic Care: Menstrual Suppression for a Gender and Sexually Diverse Patient

**DOI:** 10.15766/mep_2374-8265.11511

**Published:** 2025-04-01

**Authors:** Callie K. King, Amanda M. Ryan, Tess Chase

**Affiliations:** 1 Psychologist, Center for Behavioral Health, Johns Hopkins All Children's Hospital; 2 Third-Year Resident, Department of Obstetrics and Gynecology, Bayfront Orlando Health; 3 Physician, Department of Obstetrics and Gynecology, Maternal Fetal and Neonatal Institute, Johns Hopkins All Children's Hospital

**Keywords:** Gender Diversity, Sexual Diversity, Gender Inclusion, Diversity, Equity, Inclusion, Reproductive Health Care, Menstrual Suppression, Transgender Care, OB/GYN, Simulation, Standardized Patient

## Abstract

**Introduction:**

LGBTQ+ patients have decreased access to culturally competent gynecologic care, which contributes to health care inequity. We designed an interdisciplinary educational initiative for improving gender and sexually diverse gynecologic care among OB/GYN residents.

**Methods:**

Residents were given optional American College of Obstetricians and Gynecologists Modules on Transgender Care, a lecture about LGBTQ+ health issues, and a standardized patient simulation followed by a debrief. The case of a 25-year-old assigned female at birth (AFAB), nonbinary, pansexual patient (played by an AFAB nonbinary, pansexual individual) presented for menstrual suppression. Due to known provider discomfort in this setting, learners were assessed with postintervention surveys, rather than during the simulation, to help foster psychological safety.

**Results:**

Pre- and postsurveys assessing LGBTQ+ competence and perceived helpfulness of the training were administered to 11 residents. Statistically significant increases (*p* < .05) were observed in comfort working with LGBTQ+ patients, knowledge regarding health needs for LGBTQ+ patients, comfort discussing sexual health practices with transgender/gender diverse patients, and confidence in ability to provide resources for LGBTQ+ patients. There were positive trends in reducing assumptions of a patient's gender identity and sexual orientation (*p* = .05), confidence asking a patient's name and pronouns (*p* = .06), supervising trainees caring for gender and sexually diverse patients (*p* = .07), and comfort using inclusive language (*p* = .08).

**Discussion:**

Interdisciplinary education, including simulation, can increase resident confidence in providing gynecologic care for gender and sexually diverse patients, enhance cultural competence of providers, and help reduce inequities in LGBTQ+ gynecologic care.

## Educational Objectives

By the end of this activity, learners will be able to:

1. Demonstrate the use of appropriate language in communicating with sexual and gender diverse patients (pronouns, chosen names, sexual terminology, etc.).
2. Utilize other communication skills and best practices that foster inclusivity and affirmation with sexual and gender diverse patients (e.g., gender neutral language, trauma-informed care, appropriate and affirming language for sexual history collection).
3. Obtain a comprehensive sexual history, using the Centers for Disease Control and Prevention's 5 Ps model, and a history of hormone use with a sexual and gender diverse patient.
4. Engage in appropriate education and shared decision-making regarding contraceptive options in transgender and gender diverse patients assigned female at birth desiring amenorrhea.
5. Reflect on personal biases and prior experiences in caring for sexual and gender diverse individuals.
6. Review the epidemiology of sexual and gender diversity.
7. Identify the types of legal and policy restrictions being placed on the care of sexual and gender diverse individuals.
8. Identify resources for guidance in addressing restrictions that could impact patient care.

## Introduction

Lack of LGBTQ+ education and training for medical professionals has been highlighted in recent years due to the negative impact on quality of patient care.^1,2^ As legislation around the country has been targeting the LGBTQ+ community, especially transgender and gender diverse (TGD) individuals, medical providers are being faced with uncertainty and regulations that may further decrease comfort and confidence in providing care. This has been found to contribute to health inequities^3^ and patient avoidance of recommended or necessary medical care,^4^ especially in OB/GYN care.^5^ Physicians report feeling underprepared to work with LGBTQ+ patients and may even decline care due to fear of practicing outside their scope of competency.^6,7^

Challenges for TGD and sexually diverse patients in OB/GYN care are pervasive and expand into nearly every facet of the health care system. Lack of insurance coverage for medical gender transition (e.g., gender affirming hormone therapy, gender affirmation surgery) is a well-known phenomenon limiting access to these services.^8^ Furthermore, TGD and sexually diverse patients face challenges accessing abortion care and family planning services. There are higher incidences of unintended pregnancy, teen pregnancy, and abortion in the LGBTQ+ community when compared to their cisgender, heterosexual counterparts,^9^ with a reported 19% among TGD people who attempted to end a pregnancy on their own without clinical supervision due to perceived efficiency, lack of health insurance coverage, legal restrictions, denials of or mistreatment within clinical care, and cost.^10^ Although assisted reproductive technology has gained acceptance in more recent years, providers still have reported discomfort, and programs do not universally accept fertility preservation from and treatment of TGD patients.^11^

While education has become increasingly inclusive, gaps continue to persist. Recent literature regarding OB/GYN medical resident simulations includes only TGD patients without education on sexual diversity as well.^12,13^ A recent review of clinical education showed only 22 studies that involved TGD patient cases, of which only 15 reported specific gender identities with mostly binary transgender identities and only 10 included actors whose identities matched the patients they played.^14^ Knowledge and attention to sexual history among health care providers is lacking due to the absence of routine comprehensive and affirming discussions in training.^15^ Amongst U.S. OB/GYN residents, less than 50% felt comfortable obtaining a sexual history of a 58-year-old transgender patient planning hormone therapy and surgery and only 38% felt comfortable providing counseling, which is dramatically less than their 98% comfort in obtaining a sexual history and 97% comfort with counseling when the scenario was a cisgender 16-year-old seeking contraception.^16^

The American College of Obstetrics and Gynecology (ACOG) prioritizes provider competency in LGBTQ+ gynecologic care.^17^ Specifically, the Council on Resident Education in Obstetrics and Gynecology (CREOG)'s initiative is to

enable a diverse and inclusive environment through interpersonal communication skills with patients, other providers, students, and professionals demonstrating … an ability to create an environment of inclusivity including gender-affirming care, support of those with different abilities, support for all language preferences and medical diagnosis.^17^

Currently, ACOG provides an educational video series about TGD patient care that does not include sexually diverse gynecologic care or offer any interactive components. Although this training exists, residents in our educational initiative reported that most (64%) received <3 hours of education and had never (45%) or infrequently (55%) interacted with LGBTQ+ patients.

This publication aims to make available OB/GYN medical resident education regarding both gender and sexually diverse patients created and presented by a member of the LGBTQ+ community that utilizes an actor in the simulation who is a part of both the gender and sexually diverse communities. This low-stakes, formative simulation allows learners to build a foundation in terminology and medical knowledge using our new curriculum and solidify these concepts through experiential learning in a unique simulated patient interaction on menstrual suppression with a debrief. Similar publications in *MedEdPORTAL* include standardized cases on gender and sexual diversity in the pediatric setting, prescription of gender affirming hormone therapy, and use of inclusive language^18–20^; however, there are no publications in *MedEdPORTAL* that include cases of menstrual suppression for OB/GYN learners. Our educational initiative uses the Kirkpatrick model by fulfilling the educational objectives provided by CREOG (level 1), assessing acquired comfort with caring for gender and sexually diverse patients pre- and postintervention (level 2), and by synthesis and practice of educational concepts in a standardized patient (SP) interaction that ideally translate to patient care (level 3).

## Methods

### Implementation

This novel curriculum was developed collaboratively as an interdisciplinary team consisting of an OB/GYN physician (ally), a health service psychologist (sexually diverse), and a social worker (sexually/gender diverse). OB/GYN residents of all postgraduate year (PGY) levels (1–4) within a residency program at a teaching hospital in the southeast region of the U.S. were the audience and participants of this training. The actor playing the SP identified as a nonbinary, pansexual individual, providing authenticity and accuracy for residents to provide appropriate care for this community. The actor playing the SP wore androgynous clothing. See recruitment materials and instructions in Appendix A.

Prior to the introduction of the curriculum, OB/GYN residents were asked to read two ACOG Practice Bulletins, which are standard of care guidelines provided by the ACOG, to provide baseline knowledge specific to working with TGD patients.^21,22^ They were also asked to complete optional (approximately 1.5 hour) online training modules entitled “Improving Ob-Gyn Care for Transgender and Non-binary Individuals,” which were developed by Michigan Medicine and CREOG and are freely available on the ACOG website, as well as on YouTube.^23^ In preparation for the encounter, a curriculum consisting of a didactic lecture was presented by the psychologist. We designed the curriculum of the lecture to provide more advanced knowledge of LGBTQ+ affirming language, health disparities, typical presenting concerns and considerations for an OB/GYN visit, communication strategies, and tools to create a more affirming space for LGBTQ+ patients.

The training took place at a simulation center affiliated with the residency program. The didactic lecture was presented via a PowerPoint on a projection screen in a classroom-like space within the simulation center and utilized a combination of a visual slide deck (Appendix B) and oral instruction and discussion. The lecture was designed and delivered by author Callie King (health service psychologist) to the entire group of residents and took approximately 40 minutes.

Simulations were structured with three randomly assigned small groups of three to four residents amongst all PGYs. The small groups took turns engaging in this simulation and debriefing. During each simulation, two residents would colead the patient appointment with a focus on menstrual suppression, while the other one to two residents would join the physician and psychologist to observe. The simulation took place in a room designed as a mock physician's office with a one-way mirror for direct observation. Two-way audiovisual equipment was used to aid in assessment, but this is not necessary for the simulation. No recordings were taken. The simulation room was arranged to represent a standard clinic exam room. As the simulation did not contain a physical exam portion, the trainees and the SP were seated in chairs facing one another.

The residents were presented with information about the SP outside of the simulation room just prior to entering, including patient name, age, sex assigned at birth, and presenting concern (Appendix C). The SP was provided information about their character (Appendix D) prior to the simulation, such as legal and chosen name, pronouns, gender identity, sex assigned at birth, sexual orientation, age, presenting concerns, and fears and expectations of the visit. The SP was also given a guide for how the patient might feel at the beginning of the appointment and how those feelings might change depending on the quality of the interaction and were encouraged to incorporate these feelings into their presentation in the room and responses to the providers (Appendix A). Residents observing the simulation were instructed to watch and listen for effectiveness in regard to the educational objectives and were provided a checklist of skills (Appendix E) to assess in their observations. The simulations each lasted approximately 20 minutes.

Directly after the simulation, each small group of residents participated in a 40-minute debrief with the observing interdisciplinary health care providers. First, residents in the simulation were able to process their experience, including areas of both comfort and difficulty. Then, the SP was able to share their experience in the room with the trainees. Next, the observers were able to share strengths and areas for growth. Observing providers then provided additional information or suggestions for residents to consider, such as alternative affirming communication strategies (verbal and nonverbal) to strengthen the patient-provider relationship, legal and ethical considerations, and specific information about available menstrual suppression options. Residents were provided a handout with language examples specific to working with TGD patients (Appendix F). An example of a scripted debrief can be found in Appendix G.

### Learner Assessment

Presurveys were administered prior to the lecture, and postsurveys were administered after the simulation (Appendix H). Given that providers may be uncomfortable with this subject matter, a summative assessment was not performed to protect the learner's psychological safety. Surveys were provided voluntarily, and learners were told their engagement and responses would remain anonymous and would not impact their training. To protect the anonymity of the medical residents, no demographic information was collected, and responses were coded to match pre- and postsurveys. As no standardized measures exist for this type of data collection, we created the surveys to measure participant perceptions of knowledge, comfort, and confidence level before and after the training. Survey questions included 20 items that used 7-point Likert-type scales measuring comfort (1 = *very uncomfortable*; 7 = *very comfortable*), confidence (1 = *very unconfident*, 7 = *very confident*), and likeliness for something to happen (1 = *very likely*, 7 = *very unlikely*). For example, questions included “How comfortable do you feel overall working with gender diverse patients,” “Rate your level of confidence in asking a patient's name and pronouns,” and “Rate your level of comfort discussing sexual health practices with a gender/sexually diverse patient (e.g., use of protection, menstrual suppression, HIV PREP [preexposure prophylaxis], etc.).” The postsurveys also included three open-ended questions asking the most helpful part, the most unhelpful part, and one thing participants learned from engaging in the training.

Nonparametric Wilcoxon signed rank tests were utilized to analyze the data and assess the differences between the sample's responses on pre- and postsurveys. Themes from open-ended questions on the postsurveys about perceptions of the simulation were coded by team members and analyzed using thematic analysis. Critical action checklists were created to assess the simulated scenario's flow and educational objectives and are embedded in Appendix A. This collection of survey data was declared exempt by Orlando Health Bayfront Hospital's Institutional Review Board (case number 2085098-1 entitled Simulation Bridges Educational Gaps in Gender Diverse Gynecologic Care).

## Results

A total of 11 OB/GYN residents participated in this training. Participants included PGY levels 1 (*n* = 3), 2 (*n* = 2), 3 (*n* = 3), and 4 (*n* = 3). All participants completed both the pre- and postsurveys, resulting in a 100% response rate. Thirty-six percent of participants reported having 0 hours of previous LGBTQ+ training; 27% reported 1–2 hours; 18% reported 3–5 hours; and 18% reported more than 5 hours. Forty-five percent of participants had never seen a TGD patient before, and 55% had seen fewer than five sexually diverse patients (Table 1).

**Table 1.**
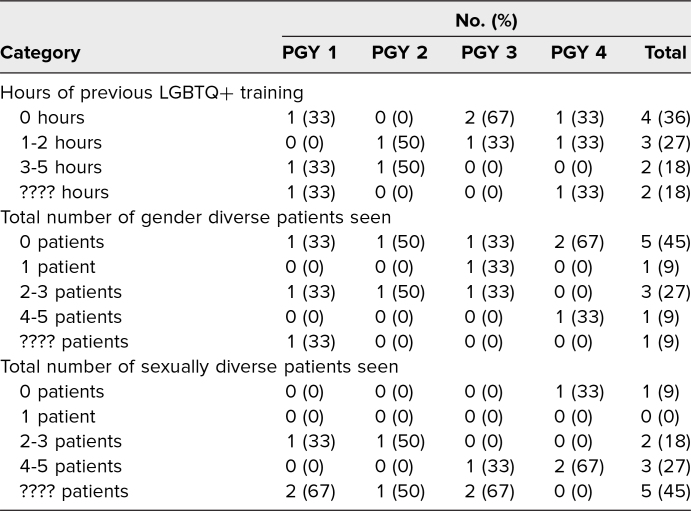
Resident LGBTQ+ Experience (*N* = 11)

Results of Wilcoxon signed rank tests revealed statistically significant increases in overall comfort in working with TGD patients (*z* = −2.2, *p* = .03, with a medium effect size [*r* = .47]), knowledge regarding specific health needs for gender and sexually diverse patients (*z* = −2.0, *p* = .05, with a medium effect size [*r* = .43]), level of comfort discussing sexual health practices with gender and sexually diverse patients (*z* = −2.6, *p* = .009, with a medium effect size [*r* = .56]), and level of confidence in ability to provide appropriate resources for gender and sexually diverse patients (*z* = −2.7, *p* = .006, with a medium effect size [*r* = .58]; Table 2).

**Table 2.**
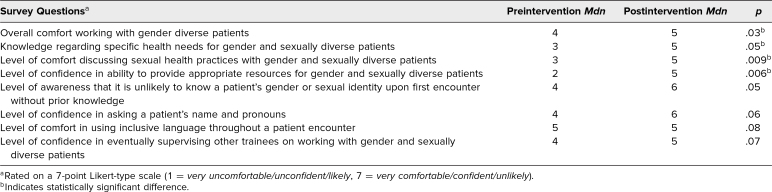
Median Score on Likert-Type Scale on Pre- and Postintervention Surveys (*N* = 11)

Additional Wilcoxon signed rank test results indicated trending (*p* < .1) increases in level of awareness that it is unlikely to know a patient's gender or sexual identity upon first encounter without prior knowledge (*z* = −1.9, *p* = .05, with a medium effect size [*r* = .40]), level of confidence in asking a patient's name and pronouns (*z* = −1.9, *p* = .06, with a medium effect size [*r* = .40]), level of comfort in using inclusive language throughout patient encounter (*z* = −1.7, *p* = .08, with a medium effect size [*r* = .39]), and level of comfort with eventually supervising other trainees on working with gender and sexually diverse patients (*z* = −1.8, *p* = .07, with a medium effect size [*r* = .39]).

Thematic analysis of open-ended questions suggested the most helpful aspects to include were the safety of the learning space (27%), discussion-based learning (45%), and the quality of the information provided (27%). Themes of how to be inclusive and safe for patients (45%), communication strategies (18%), and medical considerations specific to LGBTQ+ gynecologic care (18%) were found to be aspects learned by engaging in the training (Table 3). Participants conclusively did not find anything unhelpful regarding this training.

**Table 3.**
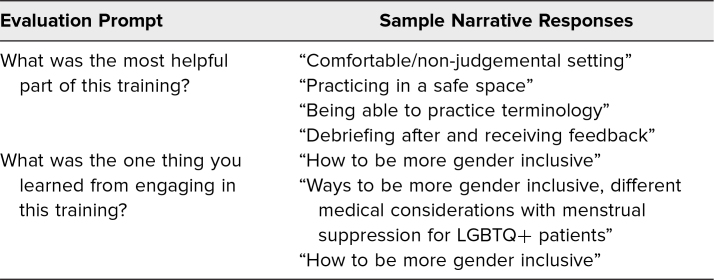
Participant Narrative Responses After Simulation Experience

## Discussion

This novel curriculum of a lecture, simulation, and debriefing specific to LGBTQ+ OB/GYN care significantly increased resident comfort working with TGD patients, discussing sexual health practices, and level of confidence in providing resources to sexual and gender diverse patients. Several trends were noted, including: increases in level of awareness that it is unlikely to know a patient's gender or sexual identity upon first encounter without prior knowledge, level of confidence in asking a patient's name and pronouns, and level of comfort with eventually supervising other trainees on working with gender and sexually diverse patients.

This curriculum has many strengths. We employed concepts from Knowles' adult learning principles and Kolb's model for experiential learning, which are superior for adult learning in medical education.^24^ Knowles’ theory states that adults use experience as the basis of learning. The simulation allowed active experimentation through a concrete experience while the debrief facilitated reflective observation and abstract conceptualization, following Kolb's model.^16^ Our curriculum development and pedagogical team consisted of members who are part of the gender and sexually diverse communities and are OB/GYN patients, which allows for authenticity and appropriate representation of these populations. This training provided a psychologically safe environment for residents to implement concepts learned.

Limitations of the curriculum include a small number of participants in a limited geographical region, which may have contributed to several findings trending positively without statistical significance and may impact generalizability. The total time of the curriculum took approximately 4 hours. While doing the simulation and debriefing in small groups is recommended to provide a safer, more comfortable environment for learners to practice new language and skills and receive feedback from others, some institutions may not have the time or resources to accommodate this structure of learning. Additionally, the use of anonymous pre and post self-assessments in this learning experience represents subjective perceptions of the residents’ abilities but is not reflective of objective or measurable skills working with diverse patients. This curriculum utilized an interdisciplinary team, including members of sexual and/or gender diverse communities, for the provision of education, as the actor playing the SP, and for coleading the debrief. Alongside this, having a health service psychologist provide the lecture and colead the debrief allowed for provision of a safe atmosphere for processing emotions and biases, asking challenging questions, and processing areas for resident improvement. While this is a strength of this curriculum, replication may be a challenge for some institutions that do not have access to such providers. In the case of non-LGBTQ+ faculty members implementing this training, we recommend pursuing continued education and use of resources on a regular basis to keep up to date with relevant literature, language, and current events impacting the LGBTQ+ community. Resources for this include the UCSF Center for Transgender Care, the GLMA (Gay and Lesbian Medical Association)/Health Professionals Advancing LGBTQ Equality, the UCLA School of Law—The Williams Institute, the Fenway Health Center, and WPATH (The World Professional Association for Transgender Health). Additionally, we encourage engagement in personal reflection on biases and privilege that may show up in this learning environment prior to participating to embody true LGBTQ+ allyship and promote a safe space for residents. Even LGBTQ+ members cannot speak for the entire community due to differences in experience, perspectives, and beliefs, so maintaining a stance of curiosity and cultural humility is important for everyone who utilizes this curriculum so as to not perpetuate stereotypes or make assumptions about individual differences of patients.

Several lessons were learned during curriculum development and implementation. Specifically, the SP will inevitably be misgendered or deadnamed, which can be a source of gender dysphoria even in a willing participant. In the name of transparency, this possibility was explicitly stated to the actor prior to the simulation and a check-in was had during and after the activity. We recommend that this be taken into consideration when recruiting SP actors. While finding a gender diverse SP was not a problem at our institution, we surmise that this could be challenging at other institutions in communities with less TGD visibility. Furthermore, not all institutions support this type of curriculum, especially in conservative areas. Legal departments may prohibit or place restrictions on implementation, such as removing institution-specific branding from educational materials or restricting access to copies/photos of the materials. Consider discussing with your institution's legal department to ensure there are no unforeseen consequences for educators or participants. Future directions include implementing this educational initiative with a larger group of learners and in different geographical regions, as well as assessing participants’ levels of comfort and confidence again after they have been able to experience LGBTQ+ patients in real-world settings.

Results of this training show a need for increased LGBTQ+ education in residency curriculum and how simulation can be a useful educational modality to achieve this goal, especially when provided by an interdisciplinary and LGBTQ+ affirming team. Ultimately, training more health care providers to deliver culturally competent LGBTQ+ gynecologic care will reduce the health inequity experienced by this population.

## Appendices


SP Recruitment Materials and Guide.docxLGBTQ+ Resident Training Lecture.pptxResident Door Entry Instructions.docxSP Case.docxChecklist for Observers.docxExample Phrases.docxScripted Debrief.docxPre- and Postsurveys.docx
*All appendices are peer reviewed as integral parts of the Original Publication.*
