# Supplementary material for: Simulation Bridges LGBTQ+ Educational Gaps in Gynecologic Care: Menstrual Suppression for a Gender and Sexually Diverse Patient
Source: MedEdPORTAL. 2025 Apr 1;21:11511. doi: 10.15766/mep_2374-8265.11511 (PMC11958776; doi:10.15766/mep_2374-8265.11511)
Supplement: Supplementary file 1 — SP Recruitment Materials and Guide.docxLGBTQ+ Resident Training Lecture.pptxResident Door Entry Instructions.docxSP Case.docxChecklist for Observers.docxExample Phrases.docxScripted Debrief.docxPre- and Postsurveys.docx [file mep_2374-8265.11511-s001.zip › G. Scripted Debrief.docx]

**Appendix G: Scripted Debrief**

Use Advocacy and Inquiry Style Debriefing. Be genuinely curious about the thought process behind certain actions so that you can elicit themes within the learner’s psychological framework to spark discussion of items not found in the scripted debrief.

Example phrases:

“I noticed that....”

“I’m concerned that....”

“Walk me through your thought process....”

Scripted Debrief

1. Can someone summarize the patient interaction?

2. What are the provider/s initial responses to the scenario?

3. Give the standardized patient actor time to reflect on the experiences they had during the scenario. Would it have been helpful for the provider/s to have approached anything differently? What did the provider/s do well? Give the gender diverse standardized patient the opportunity to speak about their real experiences and encourage a dialogue with participants.

4. What are the viewers responses to the scenario? Strengths and areas for growth for the providers?

5. What was done in this scenario that made the patient comfortable enough to open up to the provider? How can we implement that in clinical practice?

6. Are there any legal considerations regarding gender diverse care in the geographical area, especially specific to menstrual suppression? Reflect on how can we provide this evidence-based, lifesaving care within the legal limitations
