# Supplementary material for: Simulation Bridges LGBTQ+ Educational Gaps in Gynecologic Care: Menstrual Suppression for a Gender and Sexually Diverse Patient
Source: MedEdPORTAL. 2025 Apr 1;21:11511. doi: 10.15766/mep_2374-8265.11511 (PMC11958776; doi:10.15766/mep_2374-8265.11511)
Supplement: Supplementary file 1 — SP Recruitment Materials and Guide.docxLGBTQ+ Resident Training Lecture.pptxResident Door Entry Instructions.docxSP Case.docxChecklist for Observers.docxExample Phrases.docxScripted Debrief.docxPre- and Postsurveys.docx [file mep_2374-8265.11511-s001.zip › H. Pre- and Postsurveys.docx]

1. How many hours (hrs) of training prior to this simulation have you had specifically related to working with gender and sexually diverse patients?

1 2 3 4 5 6 7

0 hrs 1-2 hrs 3-5 hrs 6-8 hrs 9-10 hrs 10-20 hrs 21+ hrs

2. How relevant is gender and sexually diverse care to your practice?

1 2 3 4 5 6 7

Strongly Irrelevant Irrelevant Somewhat Irrelevant Neutral Somewhat Relevant Relevant Strongly Relevant



3. Approximately how many gender diverse patients (pts) have you seen in practice thus far?

1 2 3 4 5 6 7

0 pts 1 pt 2-3 pts 4-5 pts 5-10 pts 10-15 pts 15+ pts

4. How comfortable do you feel overall working with gender diverse patients?

1 2 3 4 5 6 7

Very uncomfortable Uncomfortable Somewhat Uncomfortable Neutral Somewhat Comfortable Comfortable Very Comfortable



5. Approximately how many sexually diverse patients (pts) have you seen in practice thus far?

1 2 3 4 5 6 7

0 pts 1 pt 2-3 pts 4-5 pts 5-10 pts 10-15 pts 15+ pts

6. How comfortable do you feel overall working with sexually diverse patients?

1 2 3 4 5 6 7

Very uncomfortable Uncomfortable Somewhat Uncomfortable Neutral Somewhat Comfortable Comfortable Very Comfortable



7. How likely are you to be able to know a patient’s gender or sexuality upon first encounter without them having told you?

1 2 3 4 5 6 7

Very Likely Likely Somewhat Likely Not Likely or Unlikely Somewhat Unlikely Unlikely Very Unlikely

8. Rate your level of confidence in knowledge regarding basic LGBTQ+ terminology:

1 2 3 4 5 6 7

Very Unconfident Unconfident Somewhat Unconfident Neutral Somewhat Confident Confident Very Confident



9. Rate your level of confidence in asking a patient’s name and pronouns:

1 2 3 4 5 6 7

Very Unconfident Unconfident Somewhat Unconfident Neutral Somewhat Confident Confident Very Confident

10. Rate your level of comfort in using inclusive language throughout a patient encounter:

1 2 3 4 5 6 7

Very uncomfortable Uncomfortable Somewhat Uncomfortable Neutral Somewhat Comfortable Comfortable Very Comfortable



11. Rate your level of comfort inquiring about a patient’s sexuality:

1 2 3 4 5 6 7

Very uncomfortable Uncomfortable Somewhat Uncomfortable Neutral Somewhat Comfortable Comfortable Very Comfortable

12. Rate your level of comfort discussing sexual history with a gender/sexually diverse patient:

1 2 3 4 5 6 7

Very uncomfortable Uncomfortable Somewhat Uncomfortable Neutral Somewhat Comfortable Comfortable Very Comfortable



13. Rate your level of confidence in knowledge regarding specific health needs for gender and sexually diverse patients:

1 2 3 4 5 6 7

Very Unconfident Unconfident Somewhat Unconfident Neutral Somewhat Confident Confident Very Confident

14. Rate your level of comfort discussing sexual health practices with a gender/sexually diverse patient (e.g., use of protection, menstrual suppression, HIV PREP, etc.):

1 2 3 4 5 6 7

Very uncomfortable Uncomfortable Somewhat Uncomfortable Neutral Somewhat Comfortable Comfortable Very Comfortable



15. Rate your level of comfort conducting a physical exam on a gender diverse patient:

1 2 3 4 5 6 7

Very uncomfortable Uncomfortable Somewhat Uncomfortable Neutral Somewhat Comfortable Comfortable Very Comfortable

16. Rate your level of comfort conducting a physical exam on a sexually diverse patient:

1 2 3 4 5 6 7

Very uncomfortable Uncomfortable Somewhat Uncomfortable Neutral Somewhat Comfortable Comfortable Very Comfortable



17. Rate your level of comfort writing notes for a gender/sexually diverse patient in an electronic medical record:

1 2 3 4 5 6 7

Very uncomfortable Uncomfortable Somewhat Uncomfortable Neutral Somewhat Comfortable Comfortable Very Comfortable

18. Rate your level of confidence in ability to provide appropriate resources for gender and sexually diverse patients:

1 2 3 4 5 6 7

Very Unconfident Unconfident Somewhat Unconfident Neutral Somewhat Confident Confident Very Confident



19. Rate your level of comfort maintaining and promoting an inclusive office environment:

1 2 3 4 5 6 7

Very uncomfortable Uncomfortable Somewhat Uncomfortable Neutral Somewhat Comfortable Comfortable Very Comfortable

20. Rate your level of comfort with eventually supervising other trainees on working with gender and sexually diverse patients:

1 2 3 4 5 6 7

Very uncomfortable Uncomfortable Somewhat Uncomfortable Neutral Somewhat Comfortable Comfortable Very Comfortable

Postsurvey

Please **circle** the number of one answer that best describes you for each of the following:

1. How helpful did you find this simulation on working with gender and sexually diverse patients?



1 2 3 4 5 6 7

Very Unhelpful Unhelpful Somewhat Unhelpful Not Helpful or Unhelpful Somewhat Helpful Helpful Very Helpful

2. How relevant is gender and sexually diverse care to your practice after this training?

1 2 3 4 5 6 7

Strongly Irrelevant Irrelevant Somewhat Irrelevant Neutral Somewhat Relevant Relevant Strongly Relevant



3. Rate how much you agree: “Engagement in this training will change the way I care for patients.”

1 2 3 4 5 6 7

Strongly Disagree Disagree Somewhat Disagree Neutral Somewhat Agree Agree Strongly Agree

4. How comfortable do you feel overall working with gender diverse patients?

1 2 3 4 5 6 7

Very uncomfortable Uncomfortable Somewhat Uncomfortable Neutral Somewhat Comfortable Comfortable Very Comfortable



5. Rate how much you agree: “Other medical residents would benefit from engaging in this training.”

1 2 3 4 5 6 7

Strongly Disagree Disagree Somewhat Disagree Neutral Somewhat Agree Agree Strongly Agree

6. How comfortable do you feel overall working with sexually diverse patients?

1 2 3 4 5 6 7

Very uncomfortable Uncomfortable Somewhat Uncomfortable Neutral Somewhat Comfortable Comfortable Very Comfortable



7. How likely are you to be able to know a patient’s gender or sexuality upon first encounter without them having told you?

1 2 3 4 5 6 7

Very Likely Likely Somewhat Likely Not Likely or Unlikely Somewhat Unlikely Unlikely Very Unlikely

8. Rate your level of confidence in knowledge regarding basic LGBTQ+ terminology:

1 2 3 4 5 6 7

Very Unconfident Unconfident Somewhat Unconfident Neutral Somewhat Confident Confident Very Confident



9. Rate your level of confidence in asking a patient’s name and pronouns:

1 2 3 4 5 6 7

Very Unconfident Unconfident Somewhat Unconfident Neutral Somewhat Confident Confident Very Confident

10. Rate your level of comfort in using inclusive language throughout a patient encounter:

1 2 3 4 5 6 7

Very uncomfortable Uncomfortable Somewhat Uncomfortable Neutral Somewhat Comfortable Comfortable Very Comfortable



11. Rate your level of comfort inquiring about a patient’s sexuality:

1 2 3 4 5 6 7

Very uncomfortable Uncomfortable Somewhat Uncomfortable Neutral Somewhat Comfortable Comfortable Very Comfortable

12. Rate your level of comfort discussing sexual history with a gender/sexually diverse patient:

1 2 3 4 5 6 7

Very uncomfortable Uncomfortable Somewhat Uncomfortable Neutral Somewhat Comfortable Comfortable Very Comfortable



13. Rate your level of confidence in your knowledge regarding specific health needs for gender and sexually diverse patients:

1 2 3 4 5 6 7

Very Unconfident Unconfident Somewhat Unconfident Neutral Somewhat Confident Confident Very Confident

14. Rate your level of comfort discussing sexual health practices with a gender/sexually diverse patient (e.g., use of protection, menstrual suppression, HIV PREP, etc.):

1 2 3 4 5 6 7

Very uncomfortable Uncomfortable Somewhat Uncomfortable Neutral Somewhat Comfortable Comfortable Very Comfortable



15. Rate your level of comfort conducting a physical exam on a gender diverse patient:

1 2 3 4 5 6 7

Very uncomfortable Uncomfortable Somewhat Uncomfortable Neutral Somewhat Comfortable Comfortable Very Comfortable

16. Rate your level of comfort conducting a physical exam on a sexually diverse patient:

1 2 3 4 5 6 7

Very uncomfortable Uncomfortable Somewhat Uncomfortable Neutral Somewhat Comfortable Comfortable Very Comfortable



17. Rate your level of comfort writing notes for a gender/sexually diverse patient in an electronic medical record:

1 2 3 4 5 6 7

Very uncomfortable Uncomfortable Somewhat Uncomfortable Neutral Somewhat Comfortable Comfortable Very Comfortable

18. Rate your level of confidence in ability to provide appropriate resources for gender and sexually diverse patients:

1 2 3 4 5 6 7

Very Unconfident Unconfident Somewhat Unconfident Neutral Somewhat Confident Confident Very Confident



19. Rate your level of comfort maintaining and promoting an inclusive office environment:

1 2 3 4 5 6 7

Very uncomfortable Uncomfortable Somewhat Uncomfortable Neutral Somewhat Comfortable Comfortable Very Comfortable

20. Rate your level of comfort with eventually supervising other trainees on working with gender and sexually diverse patients:

1 2 3 4 5 6 7

Very uncomfortable Uncomfortable Somewhat Uncomfortable Neutral Somewhat Comfortable Comfortable Very Comfortable



Please write your answers to the following questions in legible print:

21. What was the most helpful part of this training?

22. What was the most unhelpful part of this training?

23. What was one thing you learned from engaging in this training?
