# Supplementary material for: Simulation Bridges LGBTQ+ Educational Gaps in Gynecologic Care: Menstrual Suppression for a Gender and Sexually Diverse Patient
Source: MedEdPORTAL. 2025 Apr 1;21:11511. doi: 10.15766/mep_2374-8265.11511 (PMC11958776; doi:10.15766/mep_2374-8265.11511)
Supplement: Supplementary file 1 — SP Recruitment Materials and Guide.docxLGBTQ+ Resident Training Lecture.pptxResident Door Entry Instructions.docxSP Case.docxChecklist for Observers.docxExample Phrases.docxScripted Debrief.docxPre- and Postsurveys.docx [file mep_2374-8265.11511-s001.zip › A. SP Recruitment Materials and Guide.docx]

**SP Recruitment Materials and Guide**

The standardized patient needed to play this case will preferably be a member of the gender diverse community and assigned female at birth. This is needed to fulfill the role by providing authenticity and visibility to members of this community. While a well-meaning, heterosexual or cis gendered actor may want to participate, it has the potential to unintentionally reinforce biases within medical practice.

To avoid dysphoria or reactivation of previous trauma, the standardized patient must be aware of and okay with being accidentally misgendered. They may also experience feelings of dysphoria or discomfort due to any previous mistreatment by the medical community. They will be asked questions about sexual practices which may or may not feel applicable to their real life.

Authenticity is key. The standardized patient should be themselves and appear in androgynous clothing. Relying on your real experiences at the physician’s office is helpful when acting as the standardized patient. Generally speaking, most gender diverse patients are uncomfortable at the gynecologist’s office and it is okay to portray the character that way.

Case description: Alexis “Alex” Smith is a 25-year-old G0 who was assigned female at birth. The patient prefers to be called Alex. Alex is a new patient to the practice who presents to discuss menstrual suppression. The goal of this scenario is to practice using inclusive language when speaking to gender diverse patients, assess goals of reproductive care with regards to menses, contraception and childbearing, use shared decision making to create a plan that aligns with patient goals and foster a welcoming environment. Gender diverse individuals often experience inequities and discrimination within the healthcare system. This simulation is designed to expose resident physicians to an authentic scenario that incorporates knowledge of gender diverse health issues along with providing residents an opportunity to enhance interpersonal and communication skills with patients who identify as gender diverse.

***What concerns does the SP have?***

- *They are concerned about how their period affects their mood. They have a lot of anxiety in the days preceding their period and feel down once it comes. They have had to call out of work a few times and this is concerning to them because they worry it will affect their upcoming performance review. They are up for promotion to Bookstore Manager.*

***What fears does the SP have?***

- *They have never had a pap smear before and are nervous about having an exam performed. They do not want an examination today and will request to come back for screening at another time if an exam is advised. They are unwilling to have a gynecologic examination today.*

***What are the expectations or hopes that the SP has?***

- *They hope that there is a medication they can take to stop periods.*

***How is the SP feeling at the start of the scenario?***

- *They are feeling nervous and out of place at the GYN office. They will not divulge a lot of information unless directly asked or the provider makes you feel welcome and comfortable.*

***How might the SP’s feelings change during the scenario?***

- *If the provider is empathetic, welcoming and uses inclusive language, then you will relax during the scenario and open up/offer more information.*

***Information sharing:***

1. ***What information is SP expected to share with the participants during the scenario?***

- ***Bring up only if asked****: That they are interested in men and women for romantic relationships and that they may enter into a romantic relationship soon.*
- ***Spontaneously****: Periods give them a lot of anxiety and depression. They experience dysphoria with menses. After discussing menstrual suppression, they can ask about any other health maintenance requirements if pap smear screening is not brought up.*

***2. What special actions should SP perform during the scenario and when?***

- ***How should SP react/respond to specific types of questions or interactions with the learners?***
- *SP will express relief when the provider expresses empathy and uses gender inclusive language. After this happens then SP may open up and divulge more information.*
- ***What are the cues for SP to perform a specific action?***
- *If provider asks about preferred pronouns, uses inclusive language, makes SP feel welcome, then SP may open up/appear more comfortable.*
