# Supplementary material for: Simulation Bridges LGBTQ+ Educational Gaps in Gynecologic Care: Menstrual Suppression for a Gender and Sexually Diverse Patient
Source: MedEdPORTAL. 2025 Apr 1;21:11511. doi: 10.15766/mep_2374-8265.11511 (PMC11958776; doi:10.15766/mep_2374-8265.11511)
Supplement: Supplementary file 1 — SP Recruitment Materials and Guide.docxLGBTQ+ Resident Training Lecture.pptxResident Door Entry Instructions.docxSP Case.docxChecklist for Observers.docxExample Phrases.docxScripted Debrief.docxPre- and Postsurveys.docx [file mep_2374-8265.11511-s001.zip › B. LGBTQ+ Resident Training Lecture.pptx]

## Slide 1
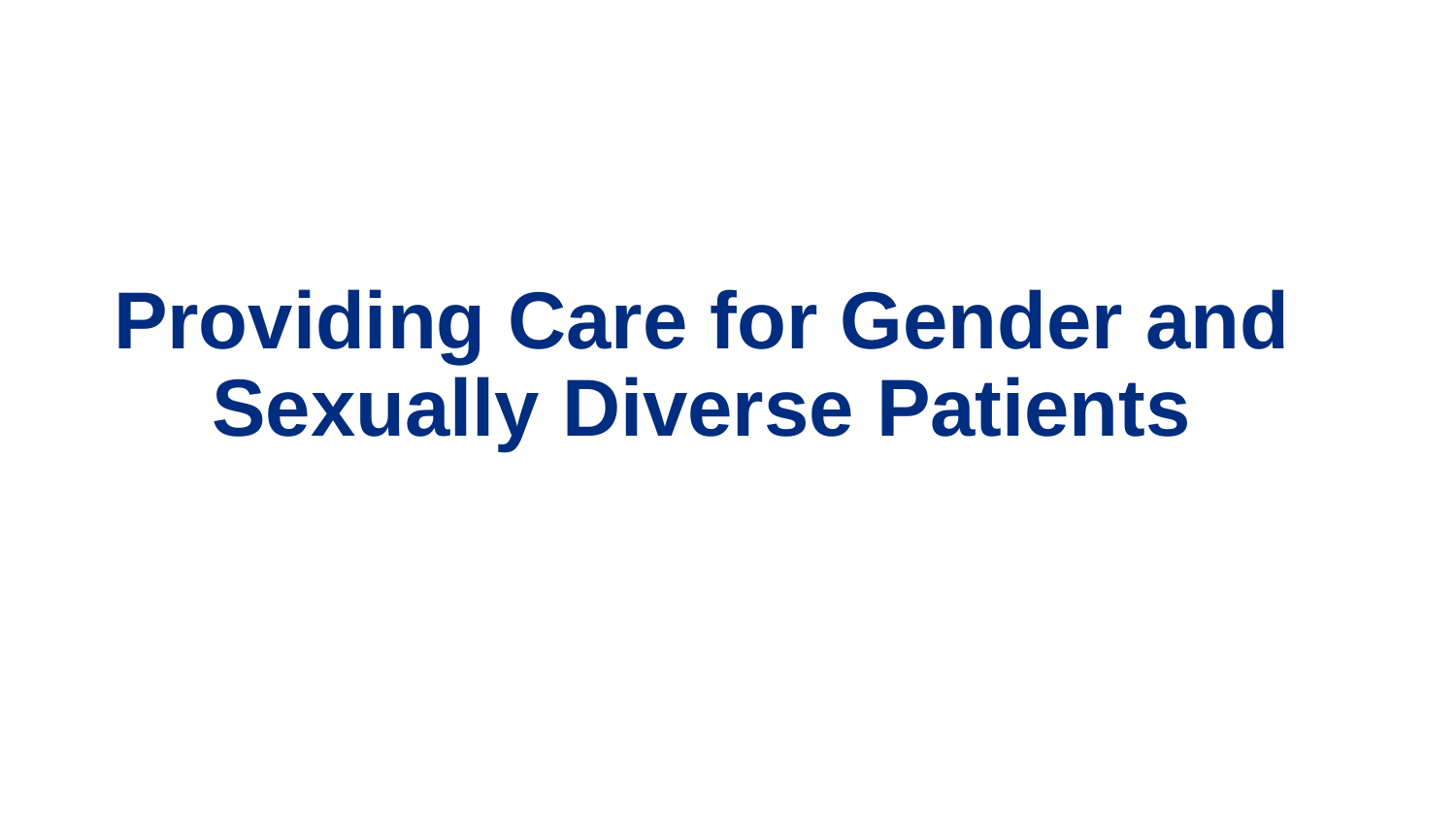

# Providing Care for Gender and Sexually Diverse Patients

## Slide 2
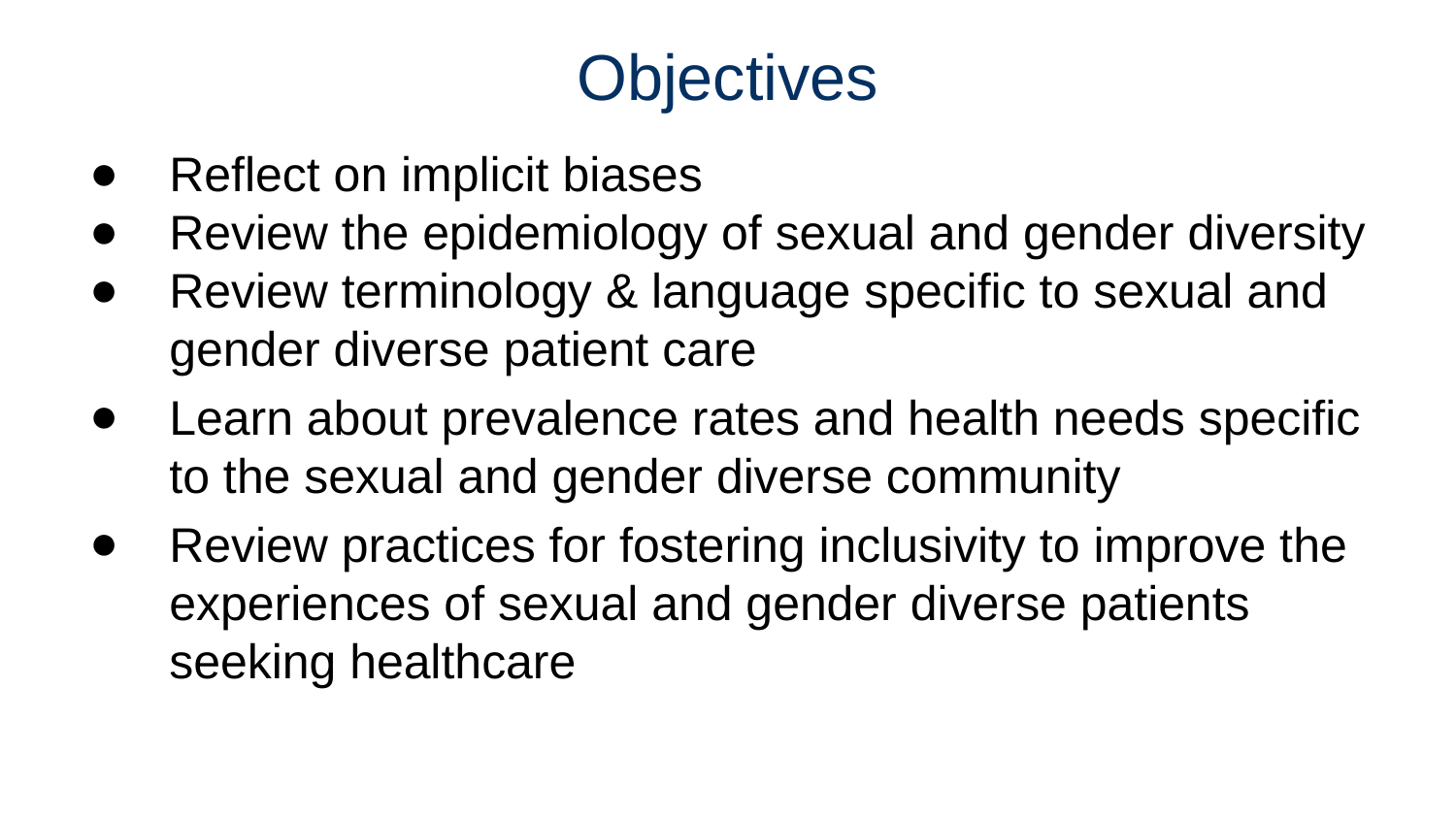

# Objectives
Reflect on implicit biases
Review the epidemiology of sexual and gender diversity
Review terminology & language specific to sexual and gender diverse patient care
Learn about prevalence rates and health needs specific to the sexual and gender diverse community
Review practices for fostering inclusivity to improve the experiences of sexual and gender diverse patients seeking healthcare

## Slide 3
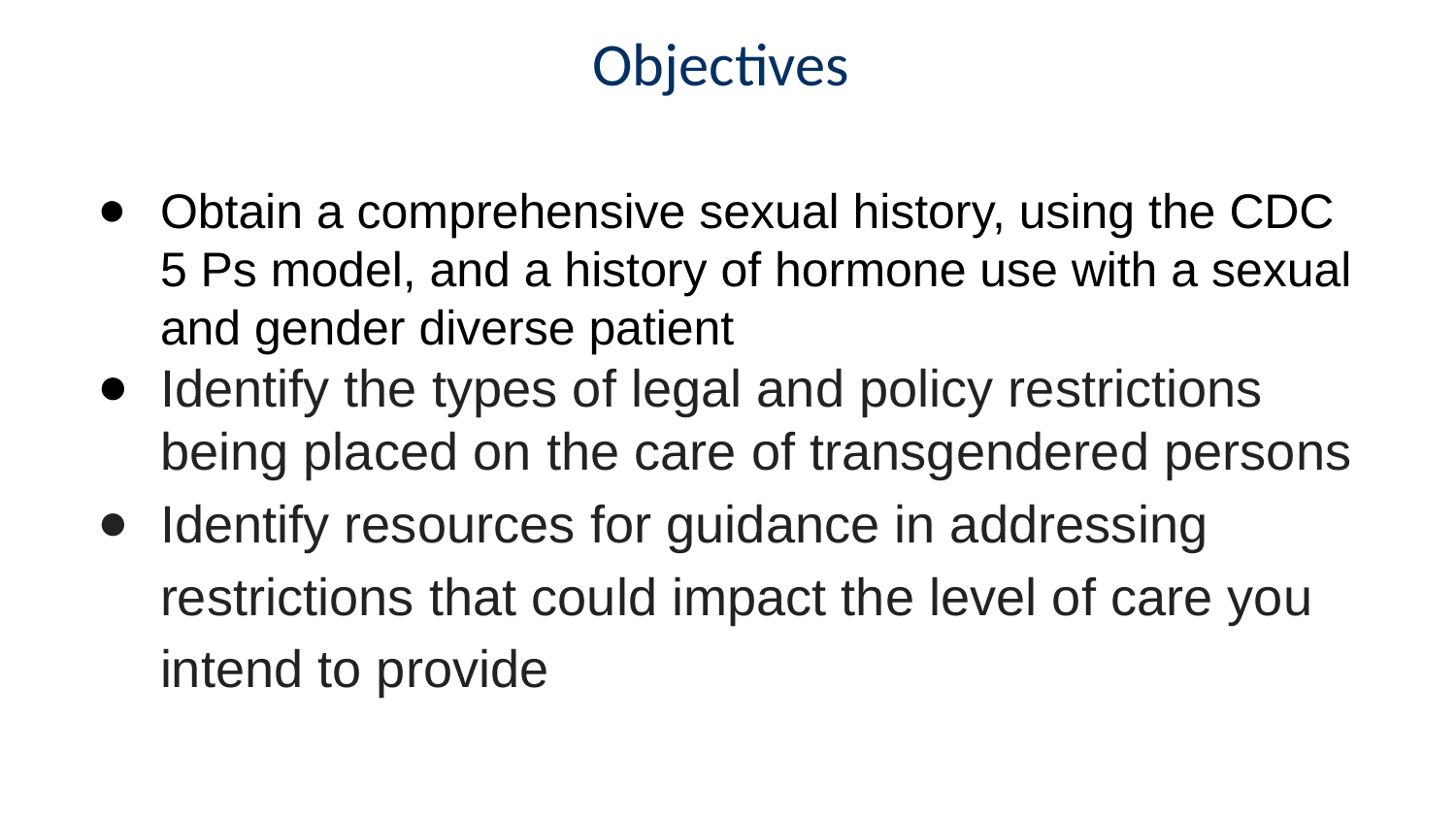

# Objectives
Obtain a comprehensive sexual history, using the CDC 5 Ps model, and a history of hormone use with a sexual and gender diverse patient
Identify the types of legal and policy restrictions being placed on the care of transgendered persons
Identify resources for guidance in addressing restrictions that could impact the level of care you intend to provide

## Slide 4
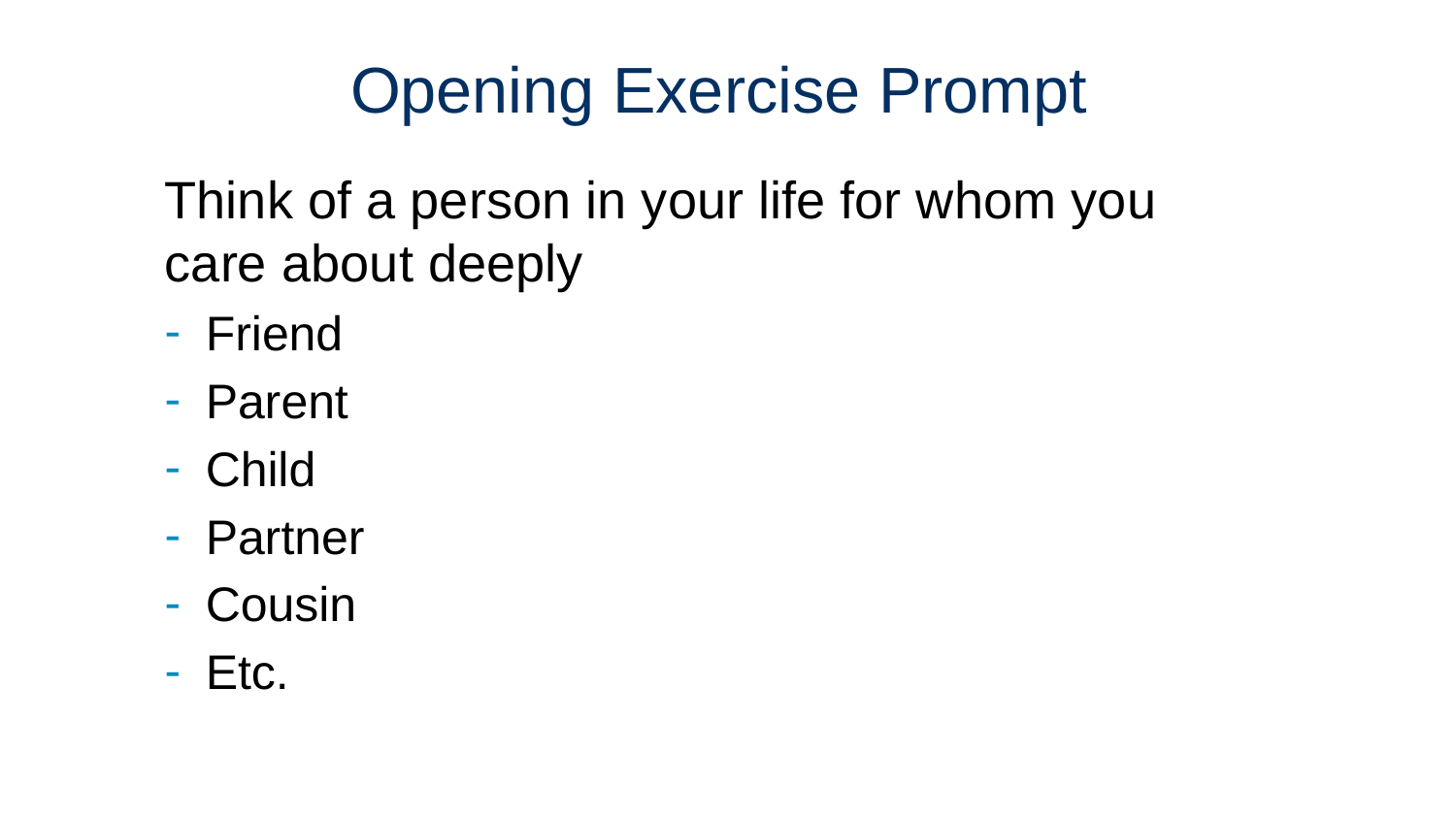

# Opening Exercise Prompt
Think of a person in your life for whom you care about deeply
Friend
Parent
Child
Partner
Cousin
Etc.

## Slide 5
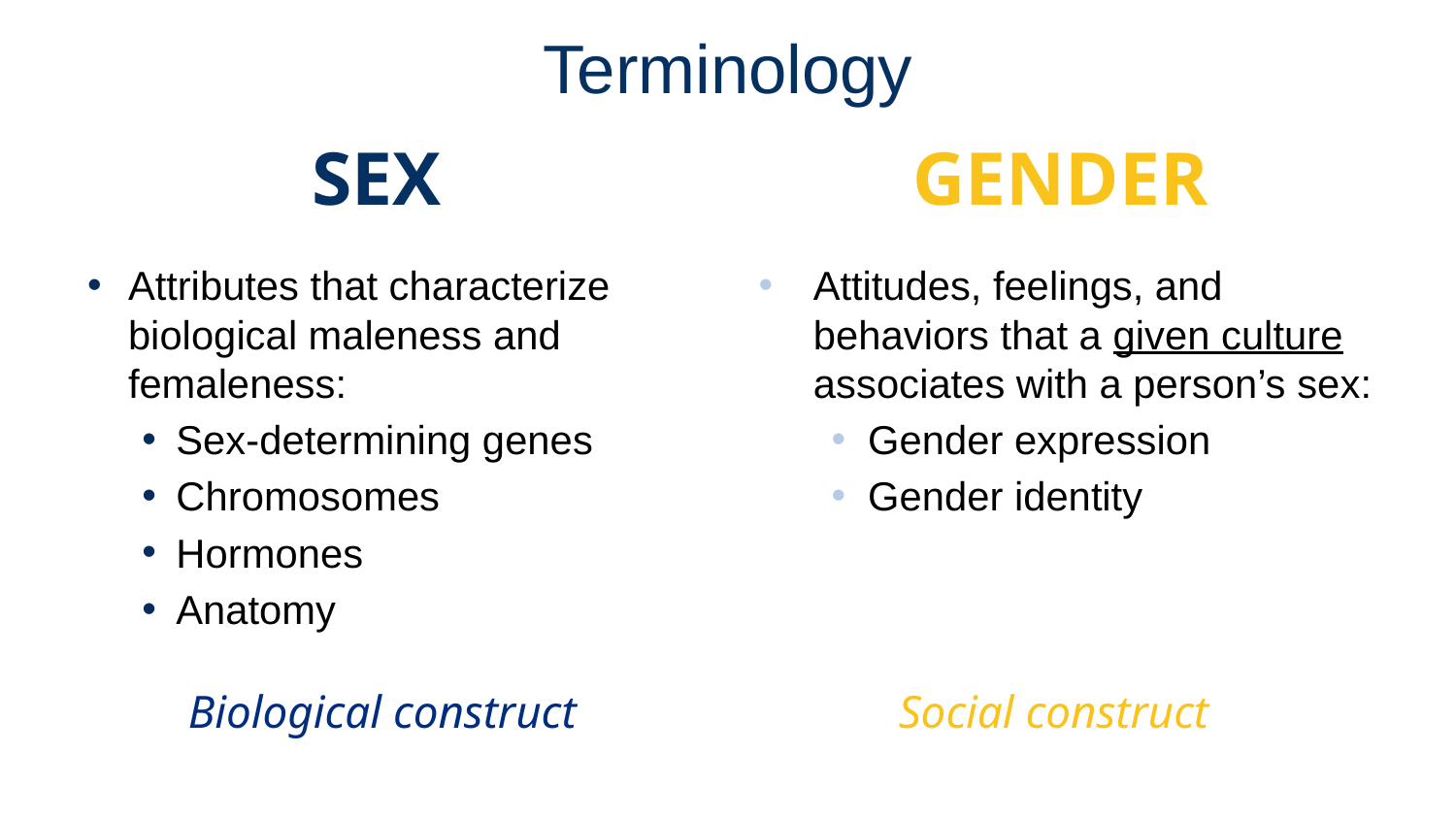

# Terminology
SEX
GENDER
Attributes that characterize biological maleness and femaleness:
Sex-determining genes
Chromosomes
Hormones
Anatomy
Attitudes, feelings, and behaviors that a given culture associates with a person’s sex:
Gender expression
Gender identity
Biological construct
Social construct

## Slide 6
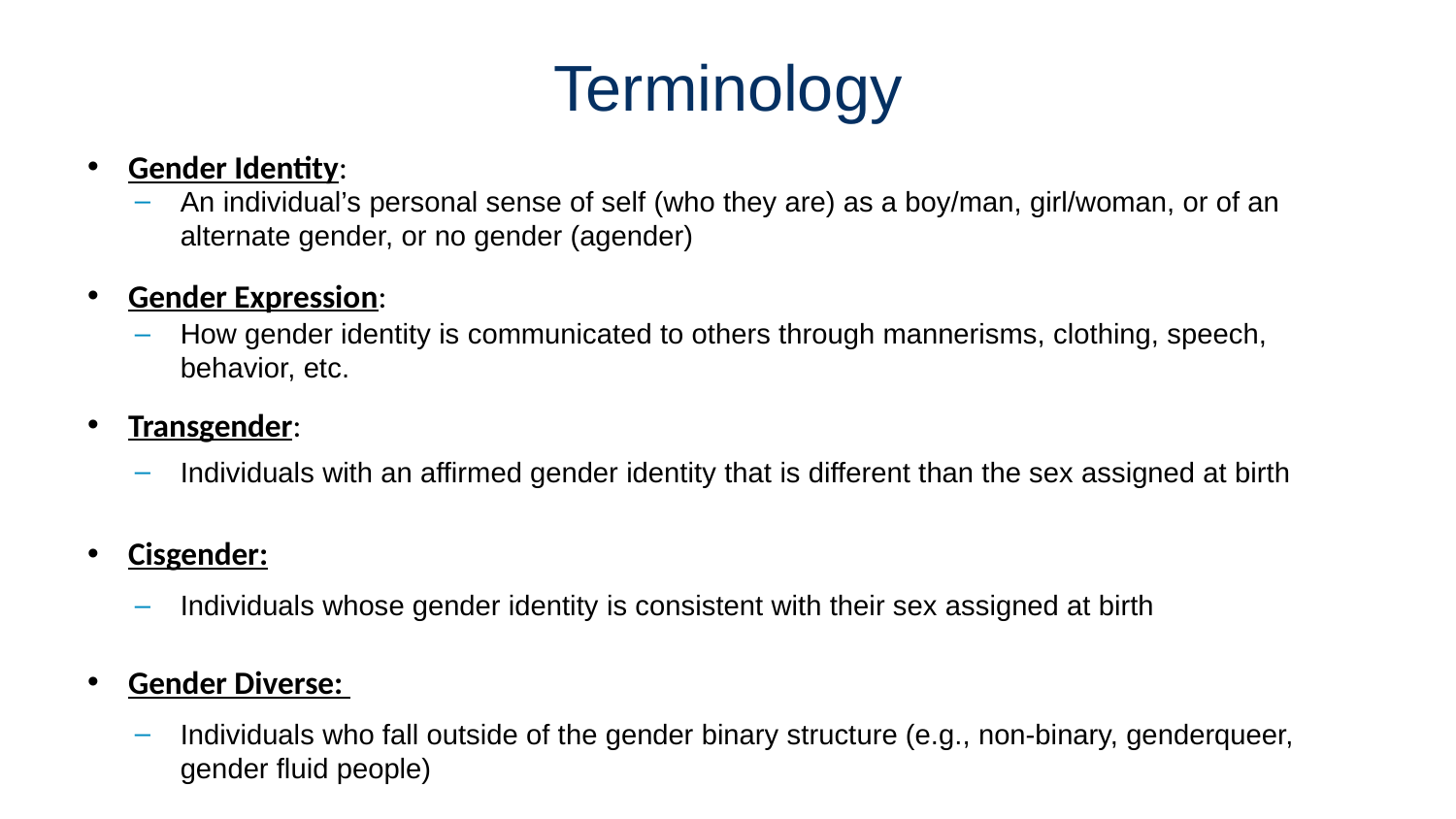

# Terminology
Gender Identity:
Gender Expression:
Transgender:
Cisgender:
Gender Diverse:
An individual’s personal sense of self (who they are) as a boy/man, girl/woman, or of an alternate gender, or no gender (agender)
How gender identity is communicated to others through mannerisms, clothing, speech, behavior, etc.
Individuals with an affirmed gender identity that is different than the sex assigned at birth
Individuals whose gender identity is consistent with their sex assigned at birth
Individuals who fall outside of the gender binary structure (e.g., non-binary, genderqueer, gender fluid people)

## Slide 7
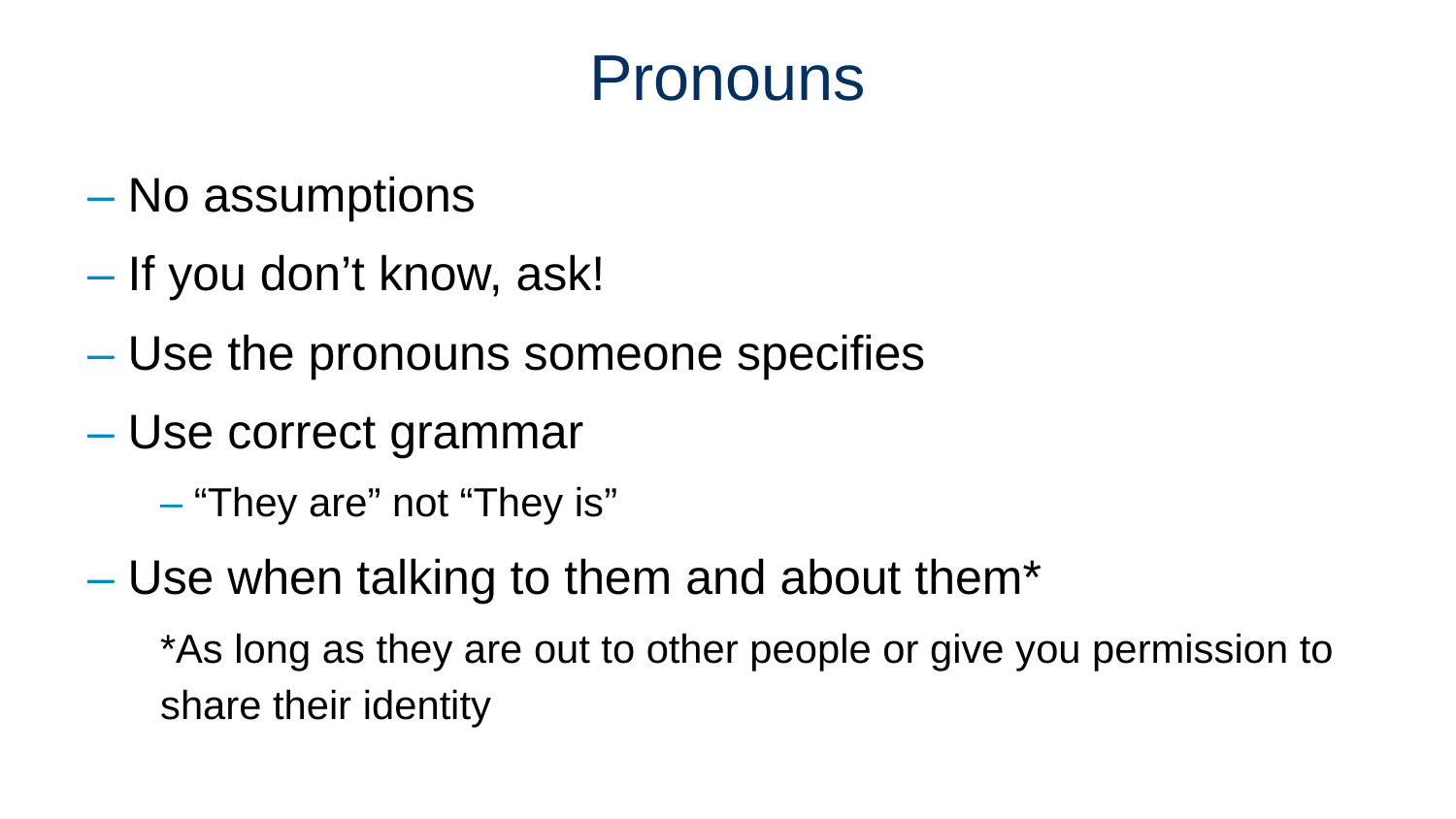

# Pronouns
‒ No assumptions
‒ If you don’t know, ask!
‒ Use the pronouns someone specifies
‒ Use correct grammar
‒ “They are” not “They is”
‒ Use when talking to them and about them*
*As long as they are out to other people or give you permission to share their identity

## Slide 8
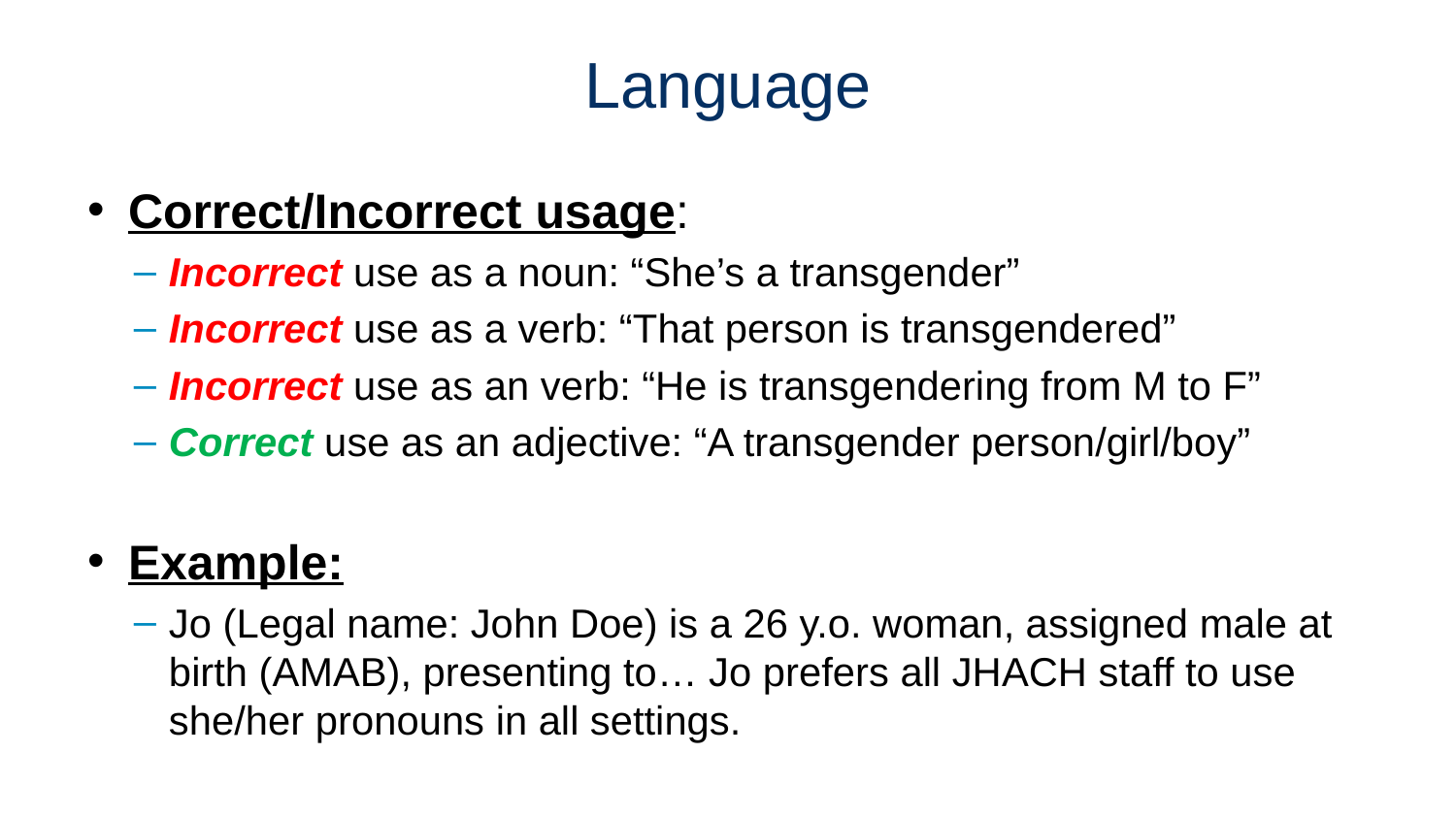

# Language
Correct/Incorrect usage:
Incorrect use as a noun: “She’s a transgender”
Incorrect use as a verb: “That person is transgendered”
Incorrect use as an verb: “He is transgendering from M to F”
Correct use as an adjective: “A transgender person/girl/boy”
Example:
Jo (Legal name: John Doe) is a 26 y.o. woman, assigned male at birth (AMAB), presenting to… Jo prefers all JHACH staff to use she/her pronouns in all settings.

## Slide 9
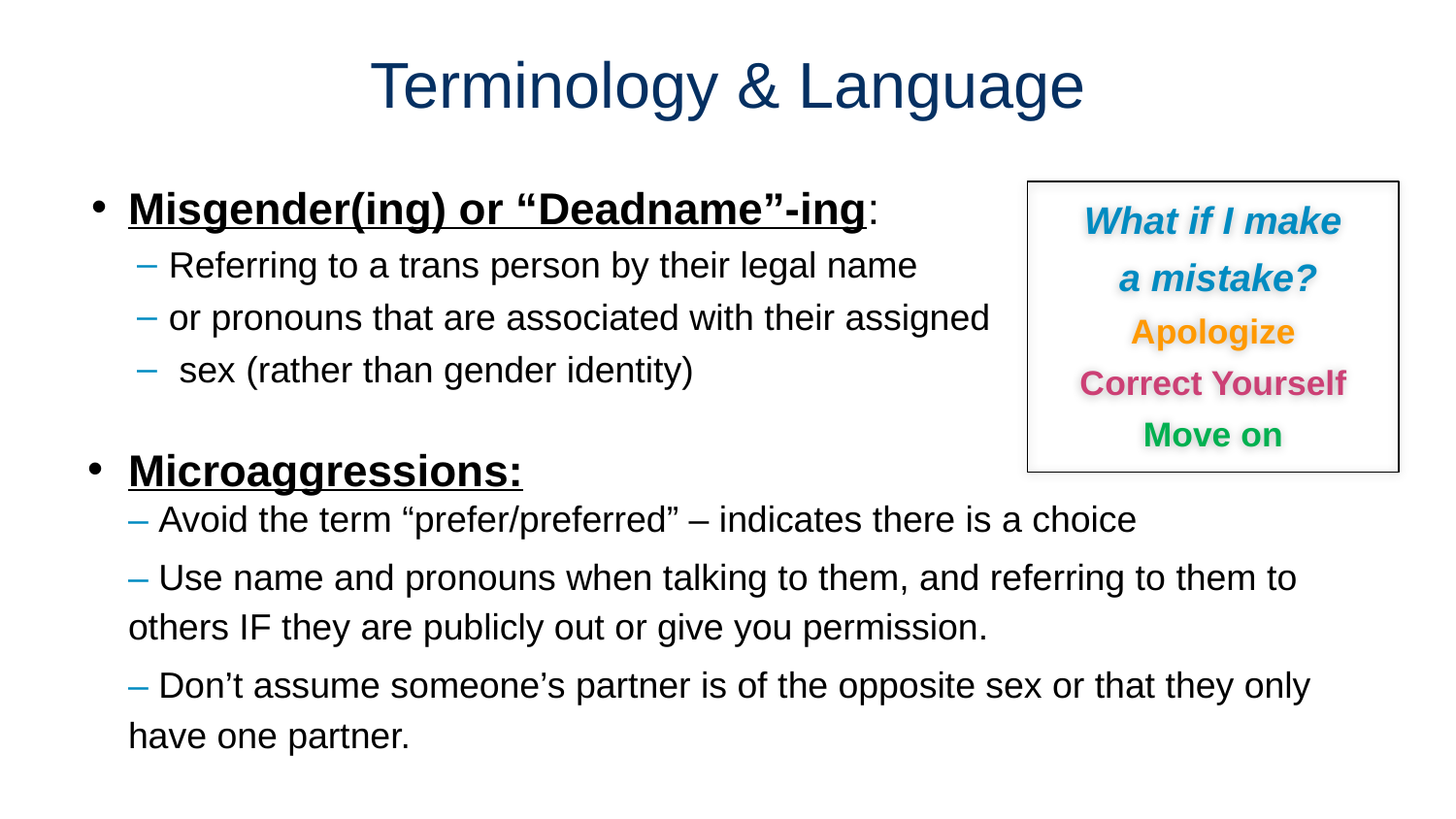

# Terminology & Language
Misgender(ing) or “Deadname”-ing:
Referring to a trans person by their legal name
or pronouns that are associated with their assigned
 sex (rather than gender identity)
Microaggressions:
– Avoid the term “prefer/preferred” – indicates there is a choice
– Use name and pronouns when talking to them, and referring to them to others IF they are publicly out or give you permission.
– Don’t assume someone’s partner is of the opposite sex or that they only have one partner.
What if I make
 a mistake?
Apologize
Correct Yourself
Move on

## Slide 10
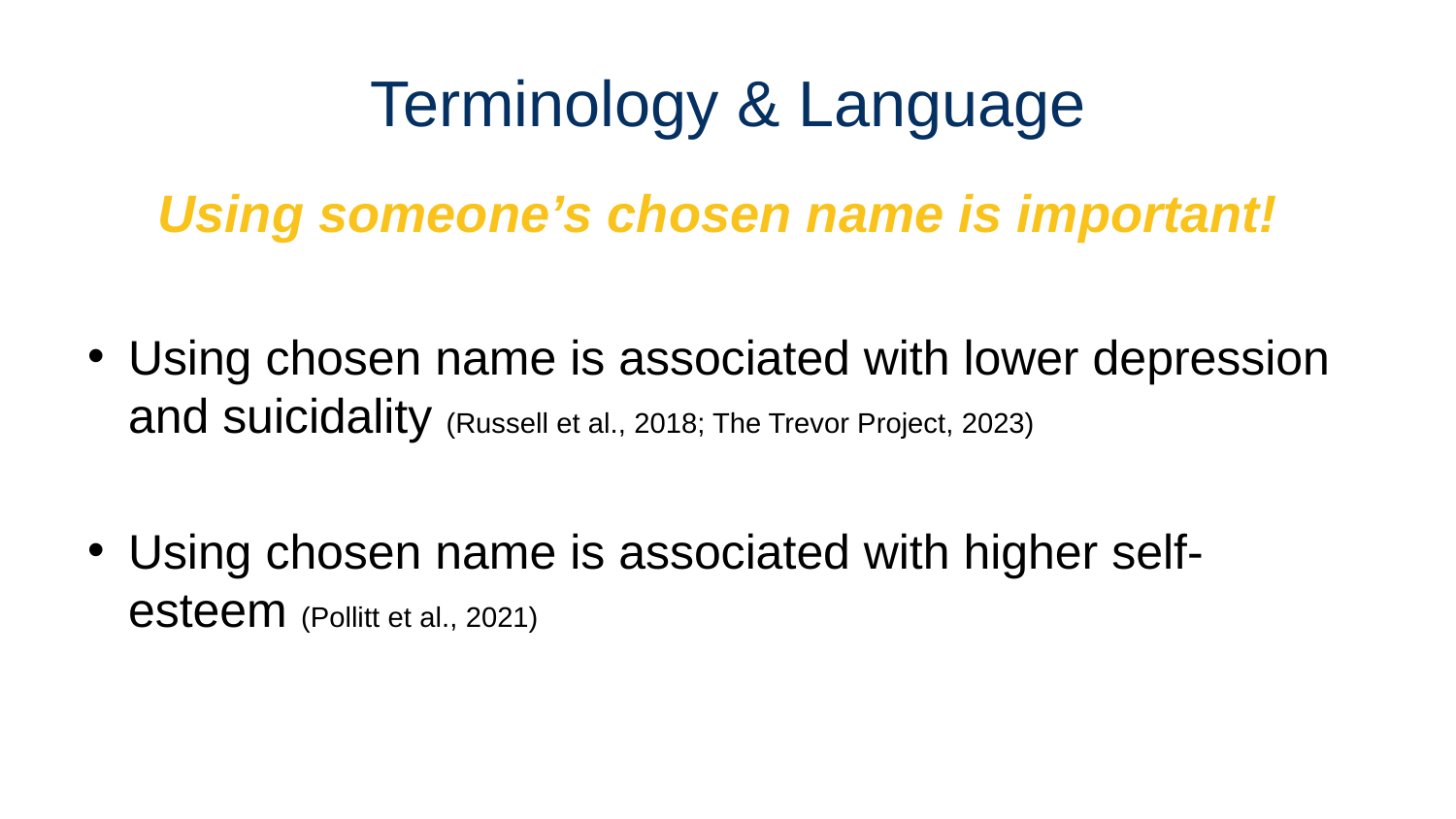

# Terminology & Language
Using someone’s chosen name is important!
Using chosen name is associated with lower depression and suicidality (Russell et al., 2018; The Trevor Project, 2023)
Using chosen name is associated with higher self-esteem (Pollitt et al., 2021)

## Slide 11
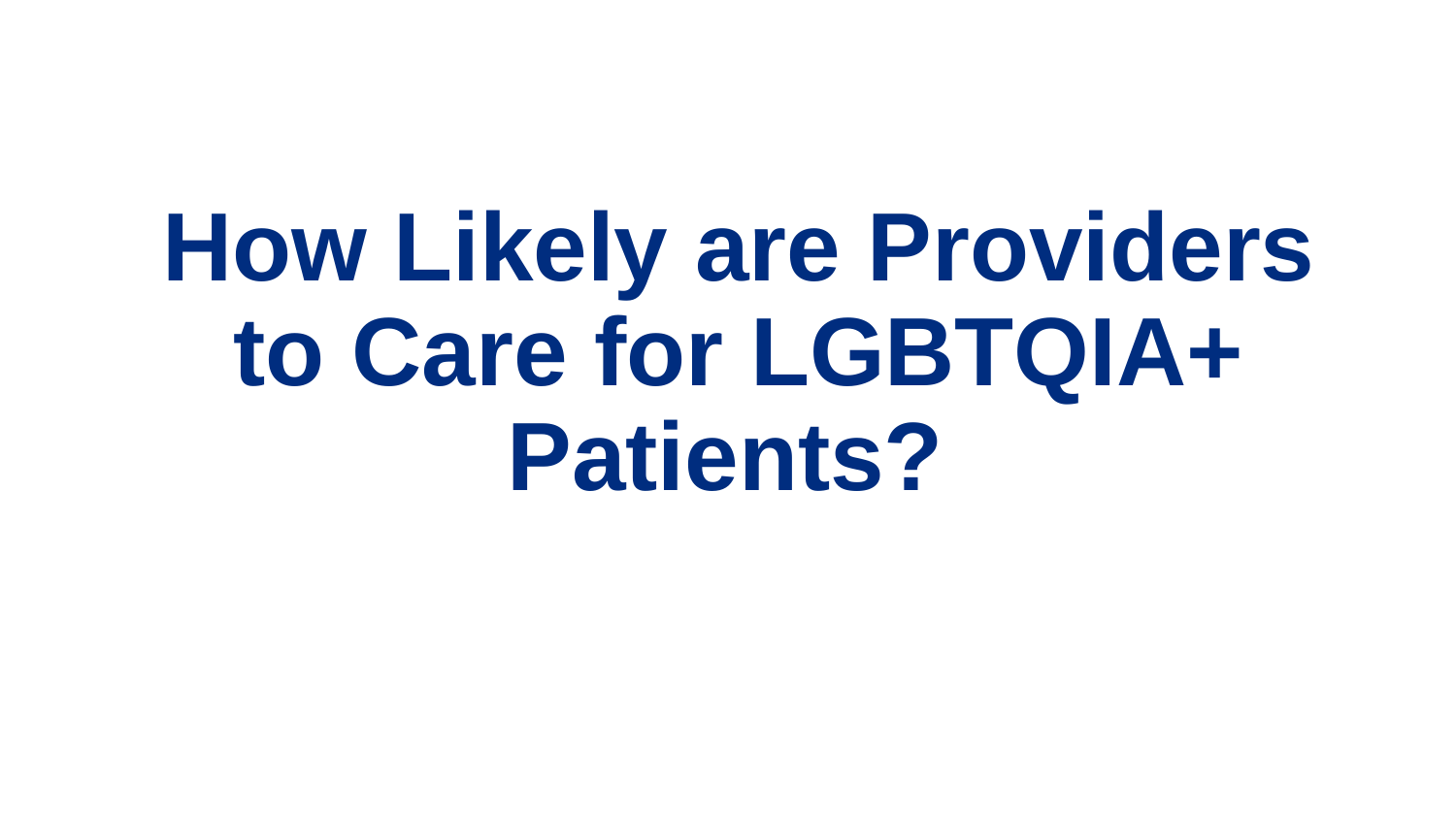

# How Likely are Providers to Care for LGBTQIA+ Patients?

## Slide 12
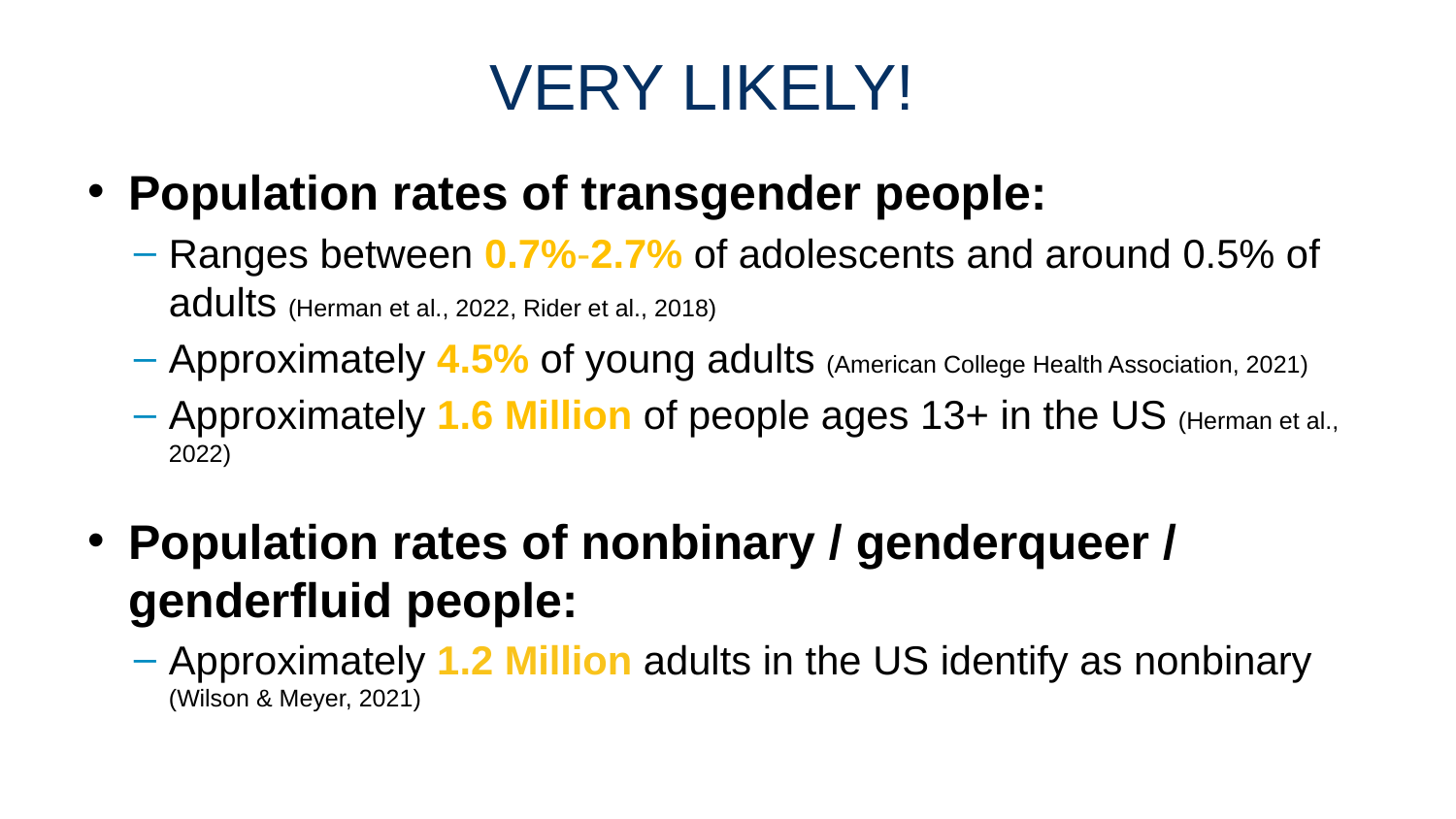

# VERY LIKELY!
Population rates of transgender people:
Ranges between 0.7%-2.7% of adolescents and around 0.5% of adults (Herman et al., 2022, Rider et al., 2018)
Approximately 4.5% of young adults (American College Health Association, 2021)
Approximately 1.6 Million of people ages 13+ in the US (Herman et al., 2022)
Population rates of nonbinary / genderqueer / genderfluid people:
Approximately 1.2 Million adults in the US identify as nonbinary (Wilson & Meyer, 2021)

## Slide 13
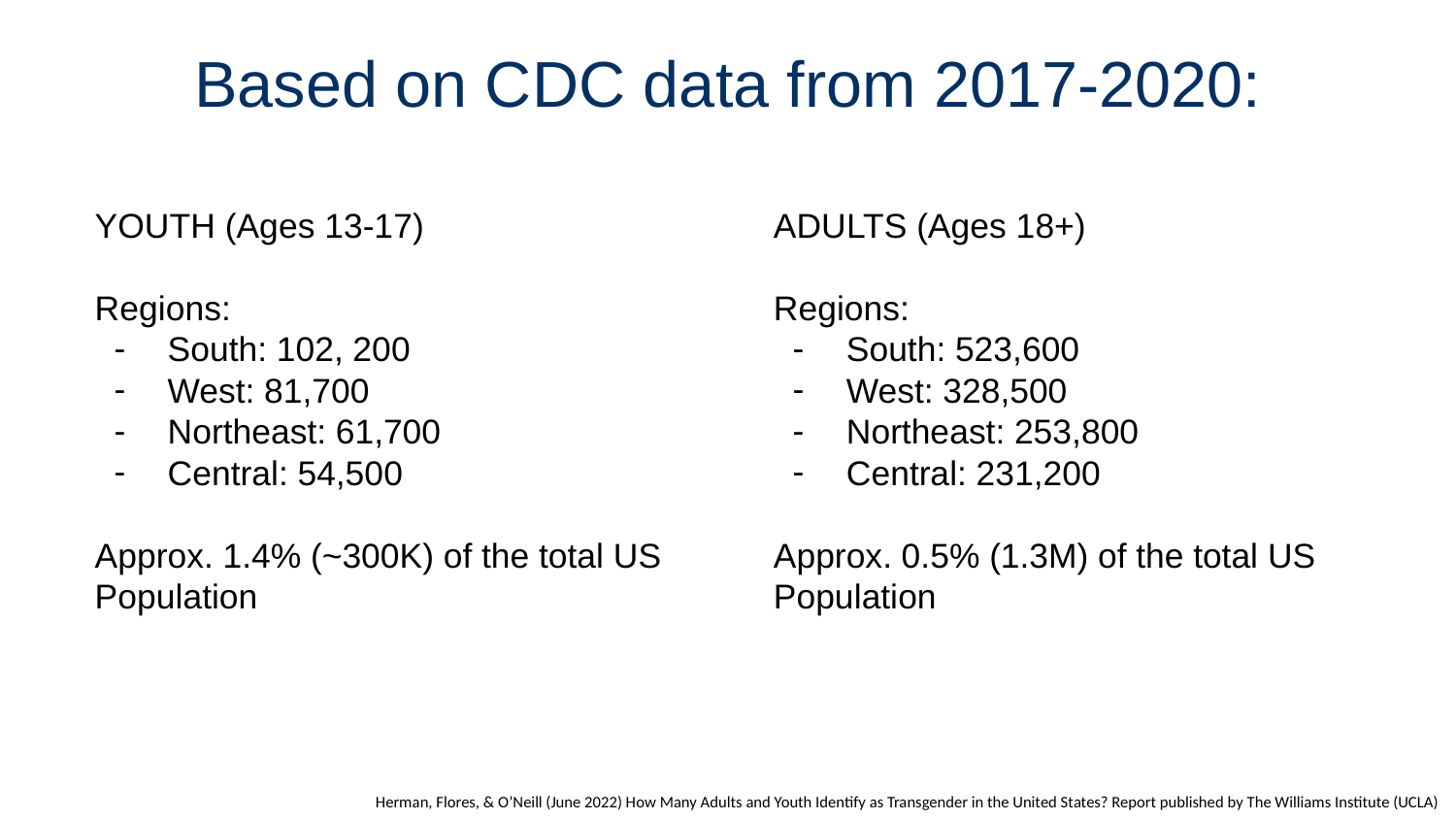

# Based on CDC data from 2017-2020:
YOUTH (Ages 13-17)
Regions:
South: 102, 200
West: 81,700
Northeast: 61,700
Central: 54,500
Approx. 1.4% (~300K) of the total US Population
ADULTS (Ages 18+)
Regions:
South: 523,600
West: 328,500
Northeast: 253,800
Central: 231,200
Approx. 0.5% (1.3M) of the total US Population
Herman, Flores, & O’Neill (June 2022) How Many Adults and Youth Identify as Transgender in the United States? Report published by The Williams Institute (UCLA)

## Slide 14
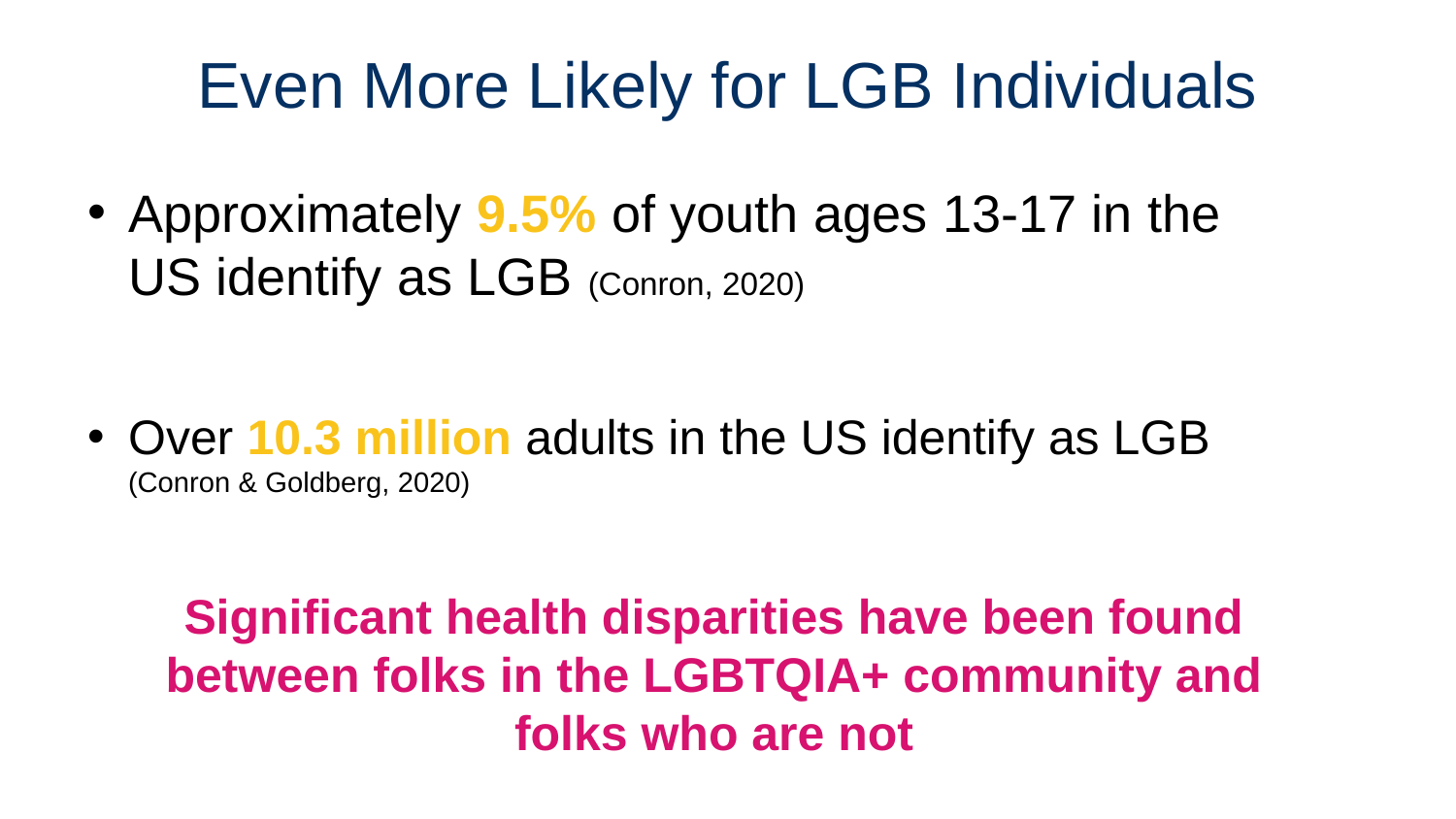

# Even More Likely for LGB Individuals
Approximately 9.5% of youth ages 13-17 in the US identify as LGB (Conron, 2020)
Over 10.3 million adults in the US identify as LGB (Conron & Goldberg, 2020)
Significant health disparities have been found between folks in the LGBTQIA+ community and folks who are not

## Slide 15
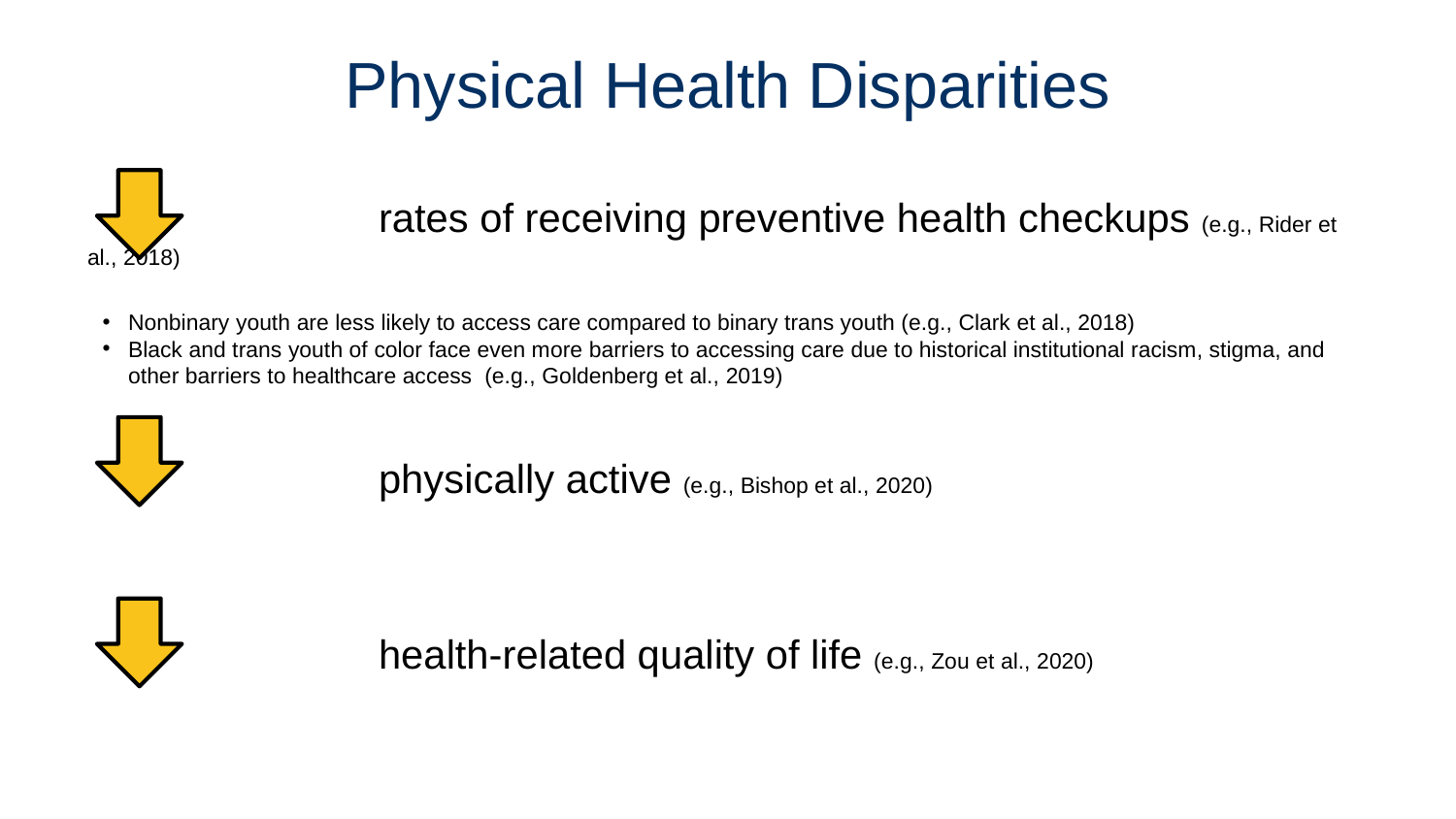

# Physical Health Disparities
		rates of receiving preventive health checkups (e.g., Rider et al., 2018)
Nonbinary youth are less likely to access care compared to binary trans youth (e.g., Clark et al., 2018)
Black and trans youth of color face even more barriers to accessing care due to historical institutional racism, stigma, and other barriers to healthcare access  (e.g., Goldenberg et al., 2019)
		physically active (e.g., Bishop et al., 2020)
		health-related quality of life (e.g., Zou et al., 2020)

## Slide 16
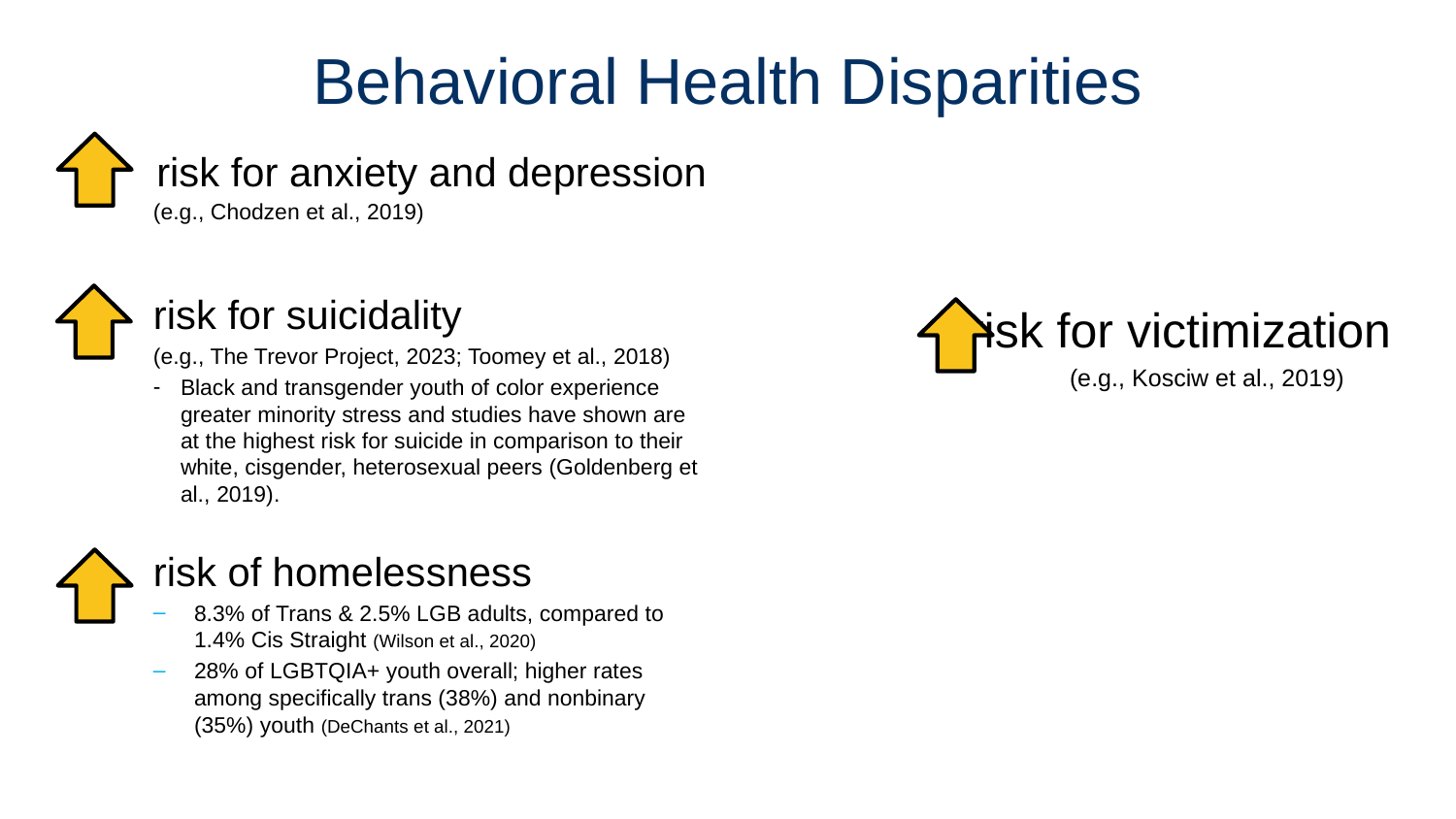

# Behavioral Health Disparities
(e.g., Chodzen et al., 2019)
risk for suicidality
(e.g., The Trevor Project, 2023; Toomey et al., 2018)
Black and transgender youth of color experience greater minority stress and studies have shown are at the highest risk for suicide in comparison to their white, cisgender, heterosexual peers (Goldenberg et al., 2019).
risk of homelessness
8.3% of Trans & 2.5% LGB adults, compared to 1.4% Cis Straight (Wilson et al., 2020)
28% of LGBTQIA+ youth overall; higher rates among specifically trans (38%) and nonbinary (35%) youth (DeChants et al., 2021)
risk for anxiety and depression
risk for victimization
	 (e.g., Kosciw et al., 2019)

## Slide 17
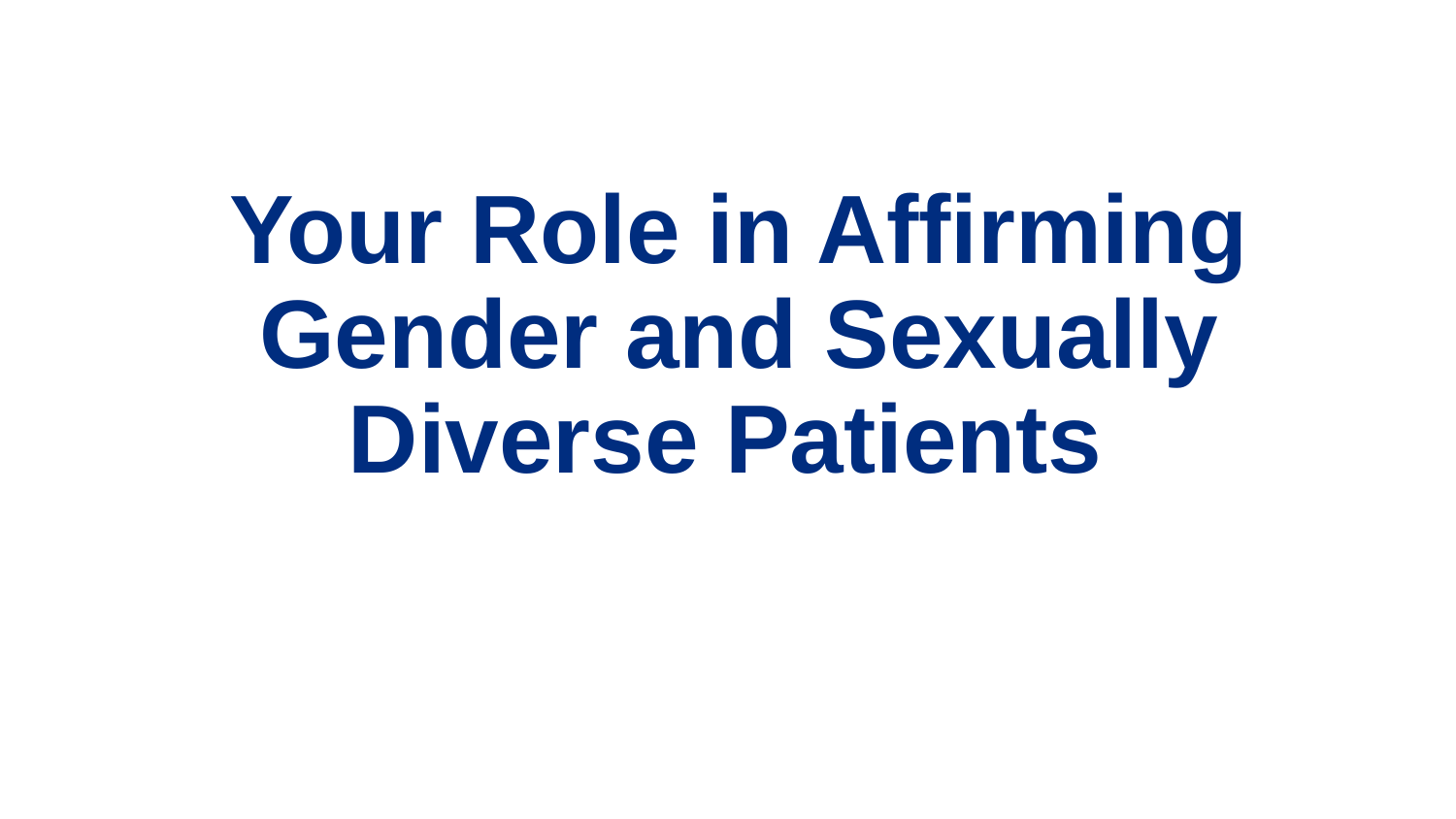

# Your Role in Affirming Gender and Sexually Diverse Patients “… the most sensitive and supportive way to interact with transgender youth is to respect their identity and use their affirmed names and pronouns”

## Slide 18
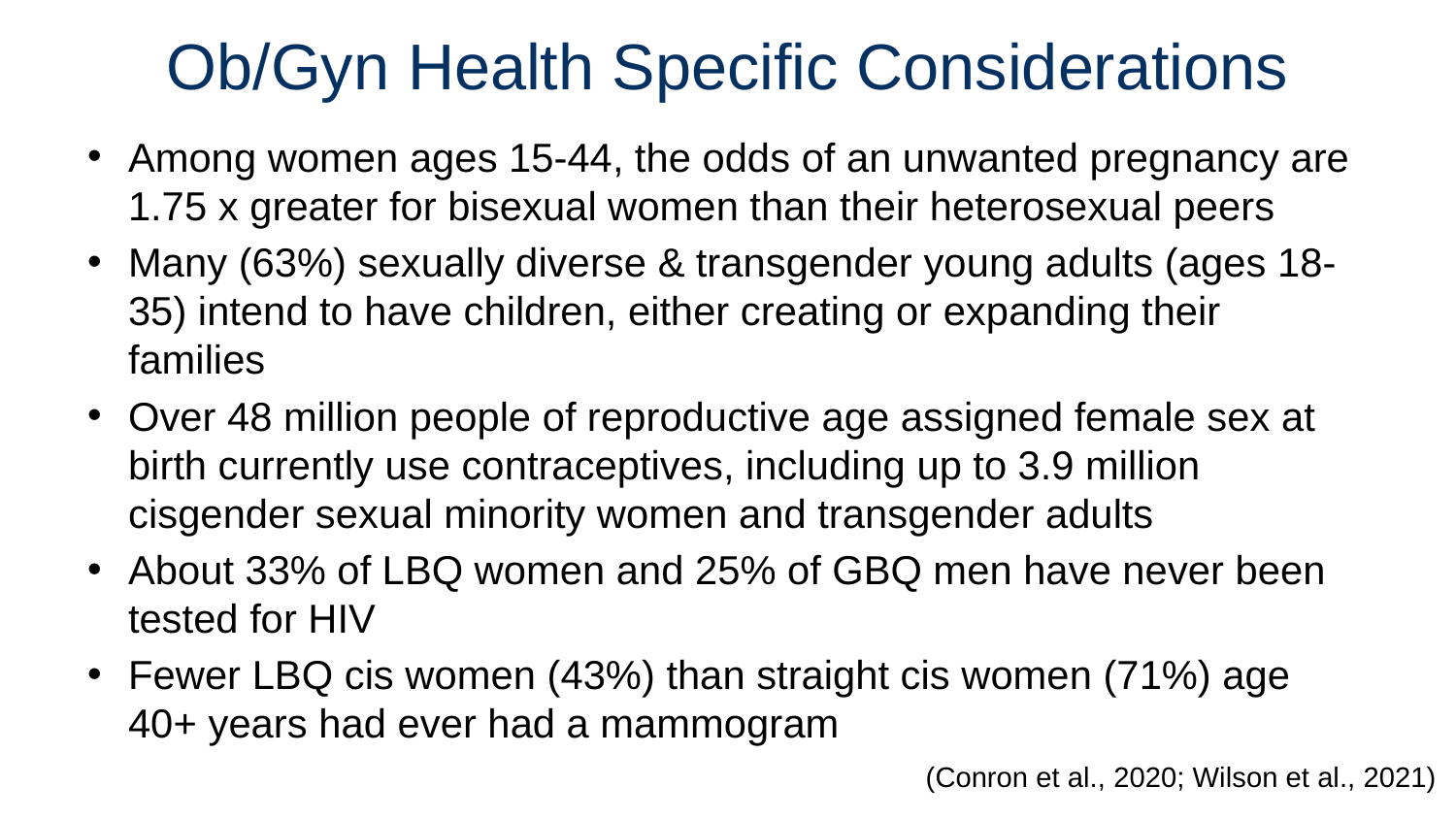

# Ob/Gyn Health Specific Considerations
Among women ages 15-44, the odds of an unwanted pregnancy are 1.75 x greater for bisexual women than their heterosexual peers
Many (63%) sexually diverse & transgender young adults (ages 18-35) intend to have children, either creating or expanding their families
Over 48 million people of reproductive age assigned female sex at birth currently use contraceptives, including up to 3.9 million cisgender sexual minority women and transgender adults
About 33% of LBQ women and 25% of GBQ men have never been tested for HIV
Fewer LBQ cis women (43%) than straight cis women (71%) age 40+ years had ever had a mammogram
(Conron et al., 2020; Wilson et al., 2021)

## Slide 19
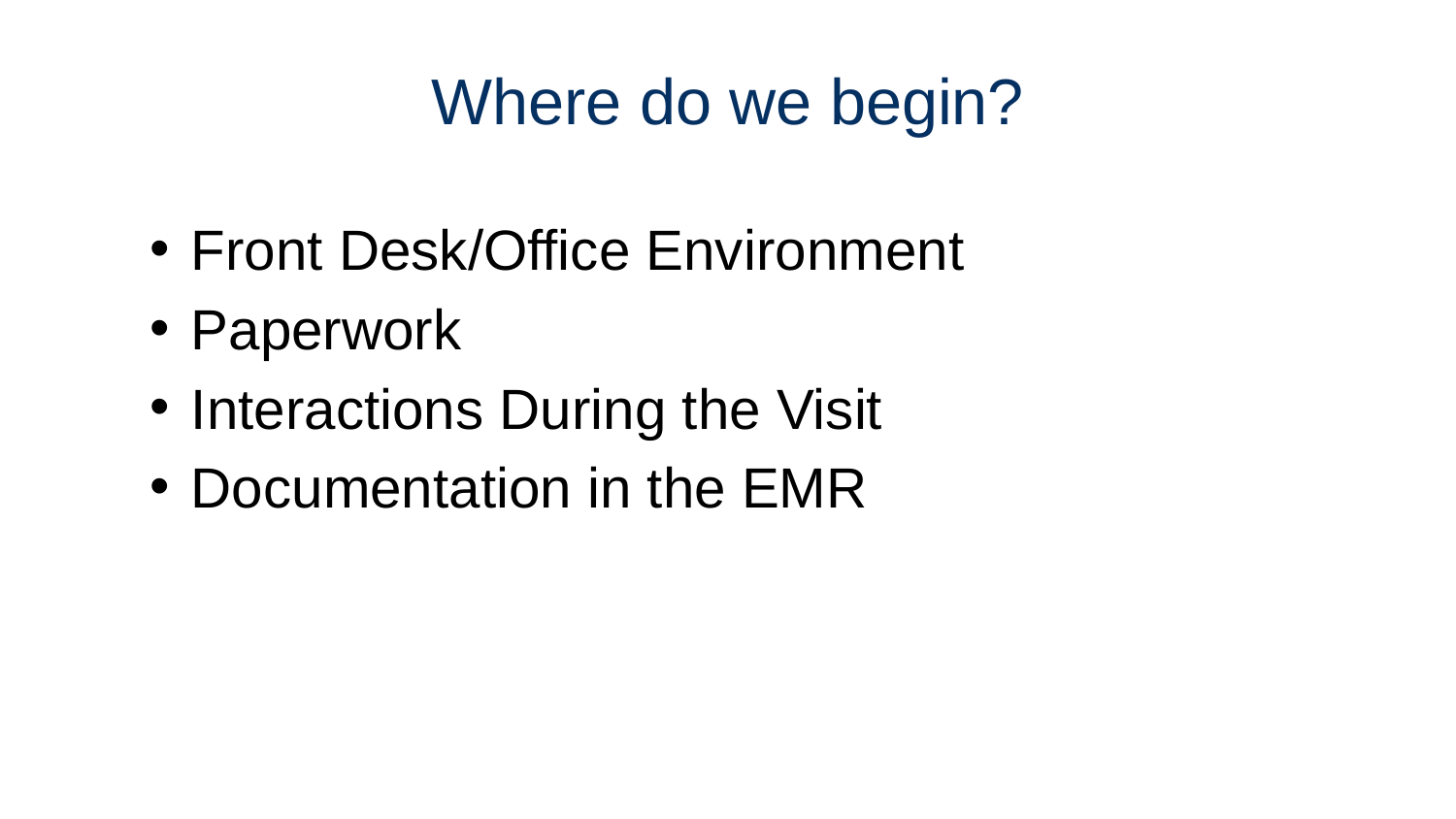

# Where do we begin?
Front Desk/Office Environment
Paperwork
Interactions During the Visit
Documentation in the EMR

## Slide 20
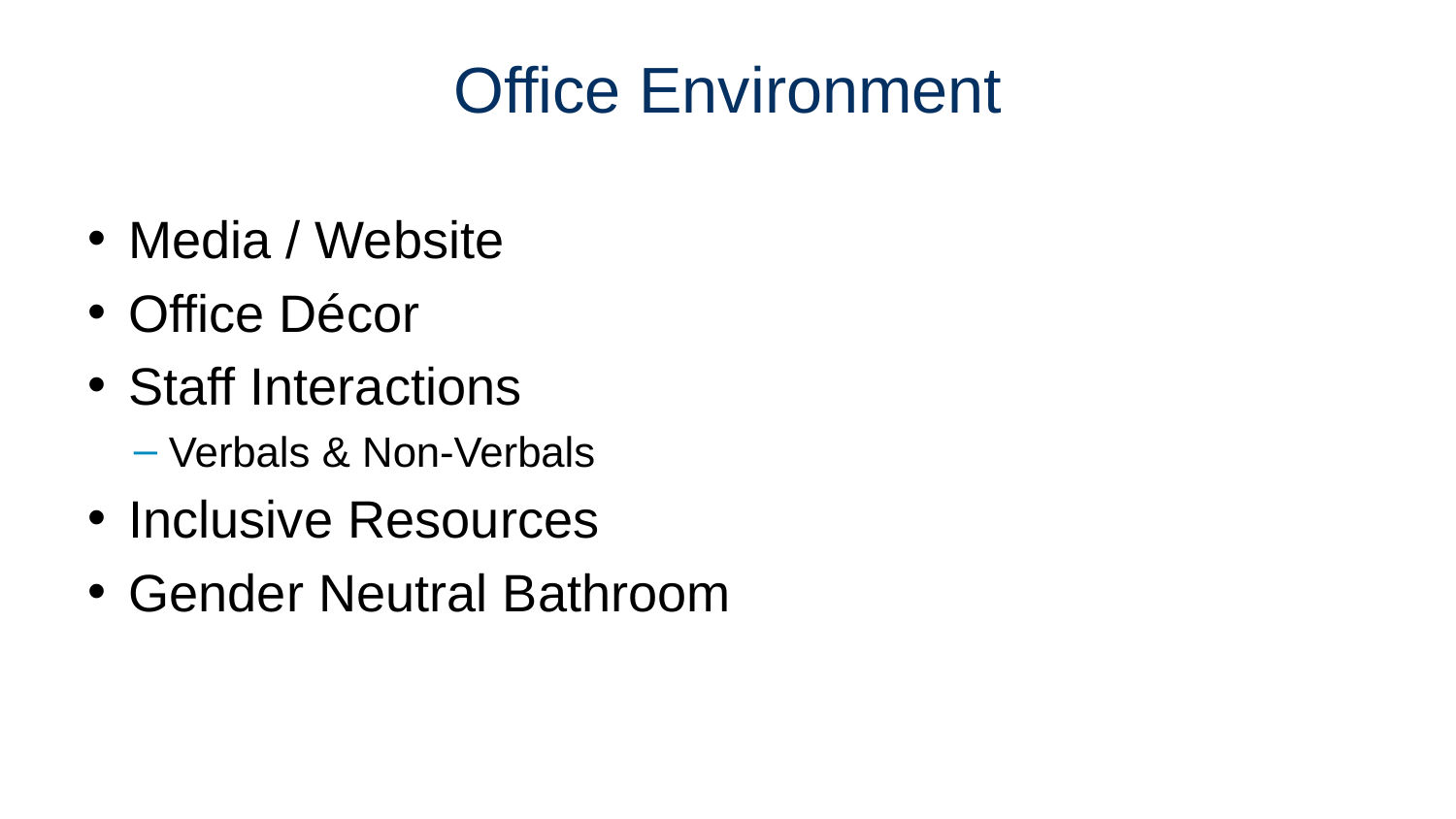

# Office Environment
Media / Website
Office Décor
Staff Interactions
Verbals & Non-Verbals
Inclusive Resources
Gender Neutral Bathroom

## Slide 21
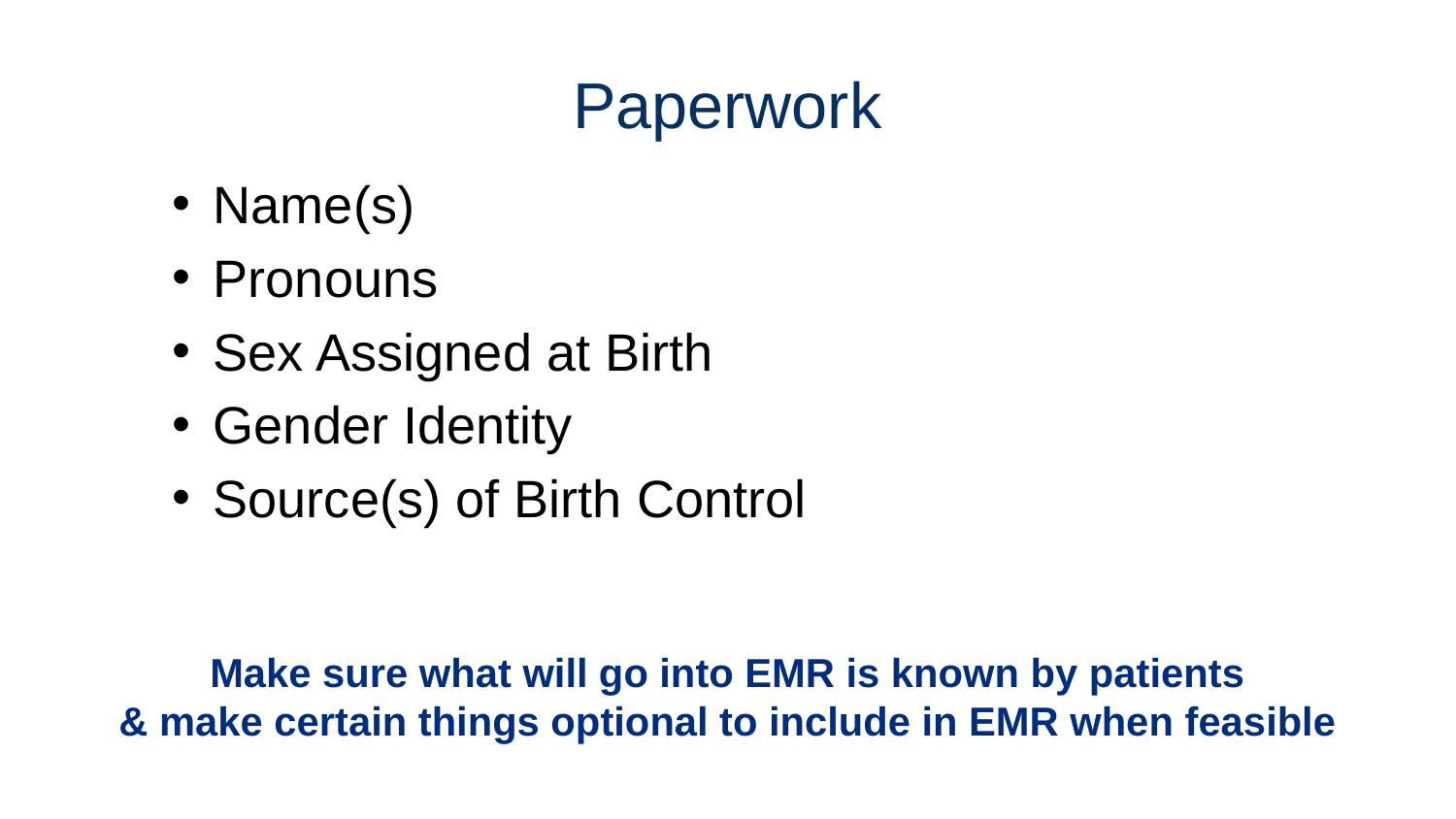

# Paperwork
Name(s)
Pronouns
Sex Assigned at Birth
Gender Identity
Source(s) of Birth Control
Make sure what will go into EMR is known by patients
& make certain things optional to include in EMR when feasible

## Slide 22
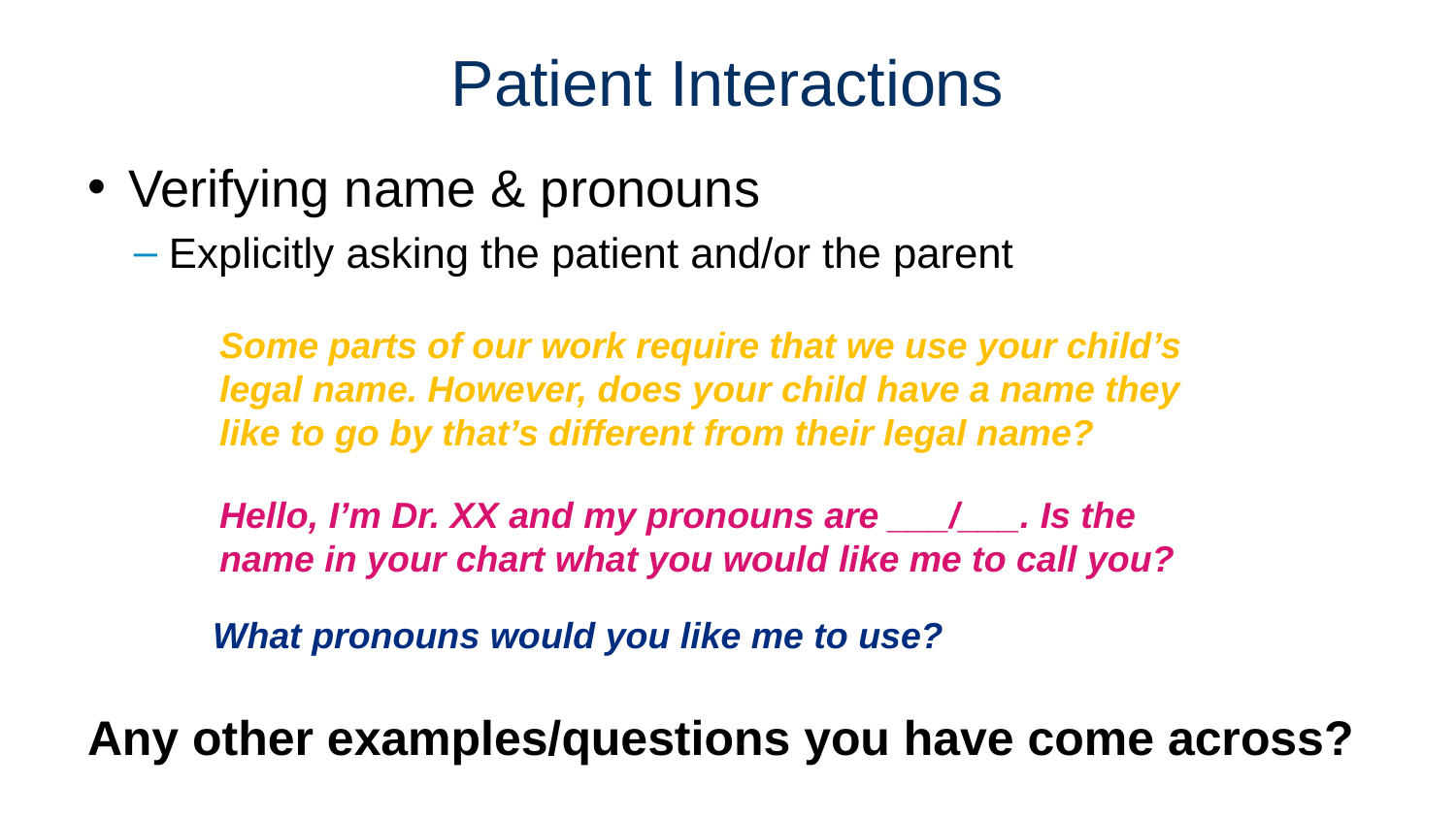

# Patient Interactions
Verifying name & pronouns
Explicitly asking the patient and/or the parent
Some parts of our work require that we use your child’s legal name. However, does your child have a name they like to go by that’s different from their legal name?
Hello, I’m Dr. XX and my pronouns are ___/___. Is the name in your chart what you would like me to call you?
What pronouns would you like me to use?
Any other examples/questions you have come across?

## Slide 23
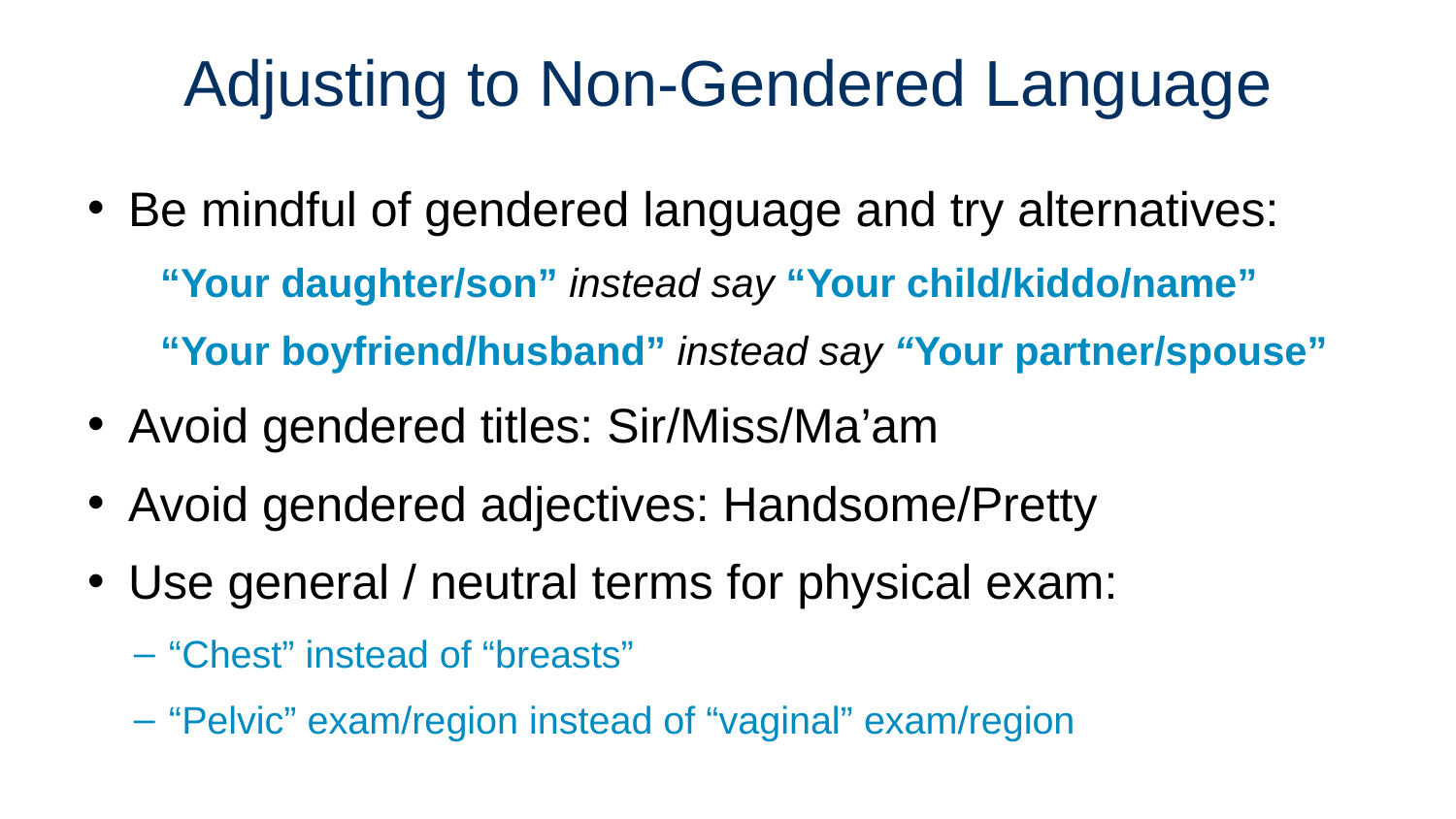

# Adjusting to Non-Gendered Language
Be mindful of gendered language and try alternatives:
“Your daughter/son” instead say “Your child/kiddo/name”
“Your boyfriend/husband” instead say “Your partner/spouse”
Avoid gendered titles: Sir/Miss/Ma’am
Avoid gendered adjectives: Handsome/Pretty
Use general / neutral terms for physical exam:
“Chest” instead of “breasts”
“Pelvic” exam/region instead of “vaginal” exam/region

## Slide 24
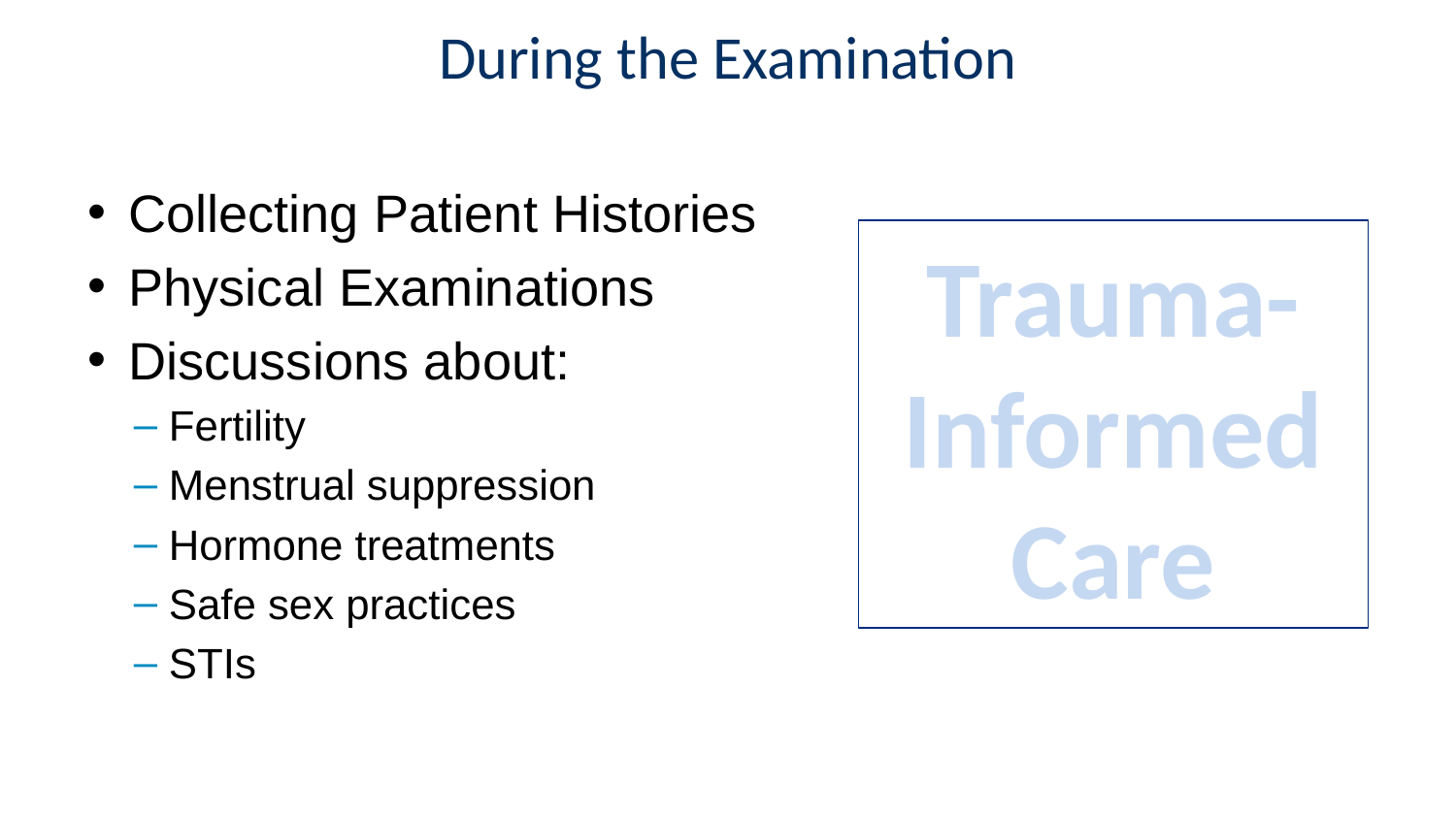

# During the Examination
Collecting Patient Histories
Physical Examinations
Discussions about:
Fertility
Menstrual suppression
Hormone treatments
Safe sex practices
STIs
Trauma-
Informed
Care

## Slide 25
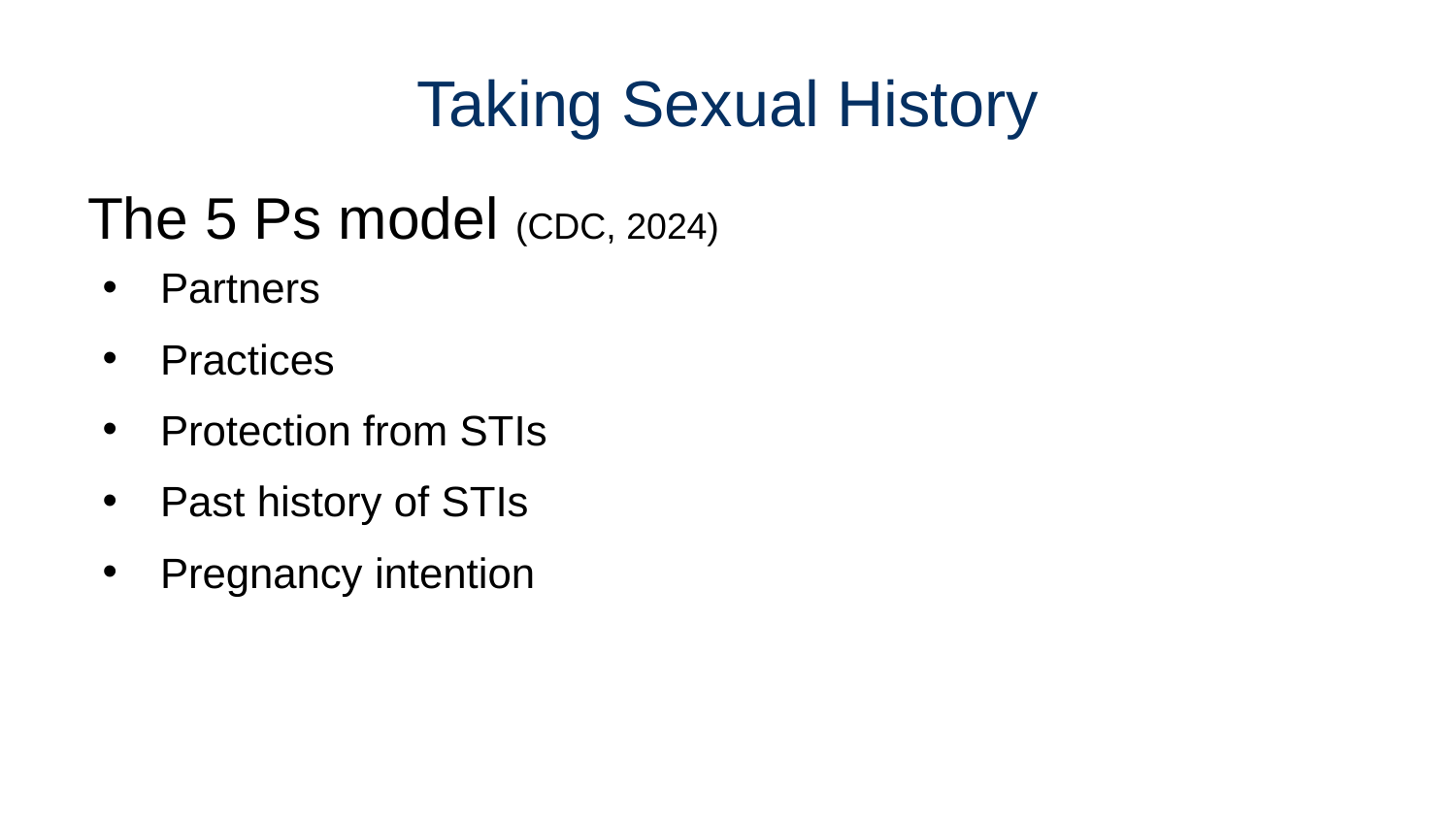

# Taking Sexual History
The 5 Ps model (CDC, 2024)
Partners
Practices
Protection from STIs
Past history of STIs
Pregnancy intention

## Slide 26
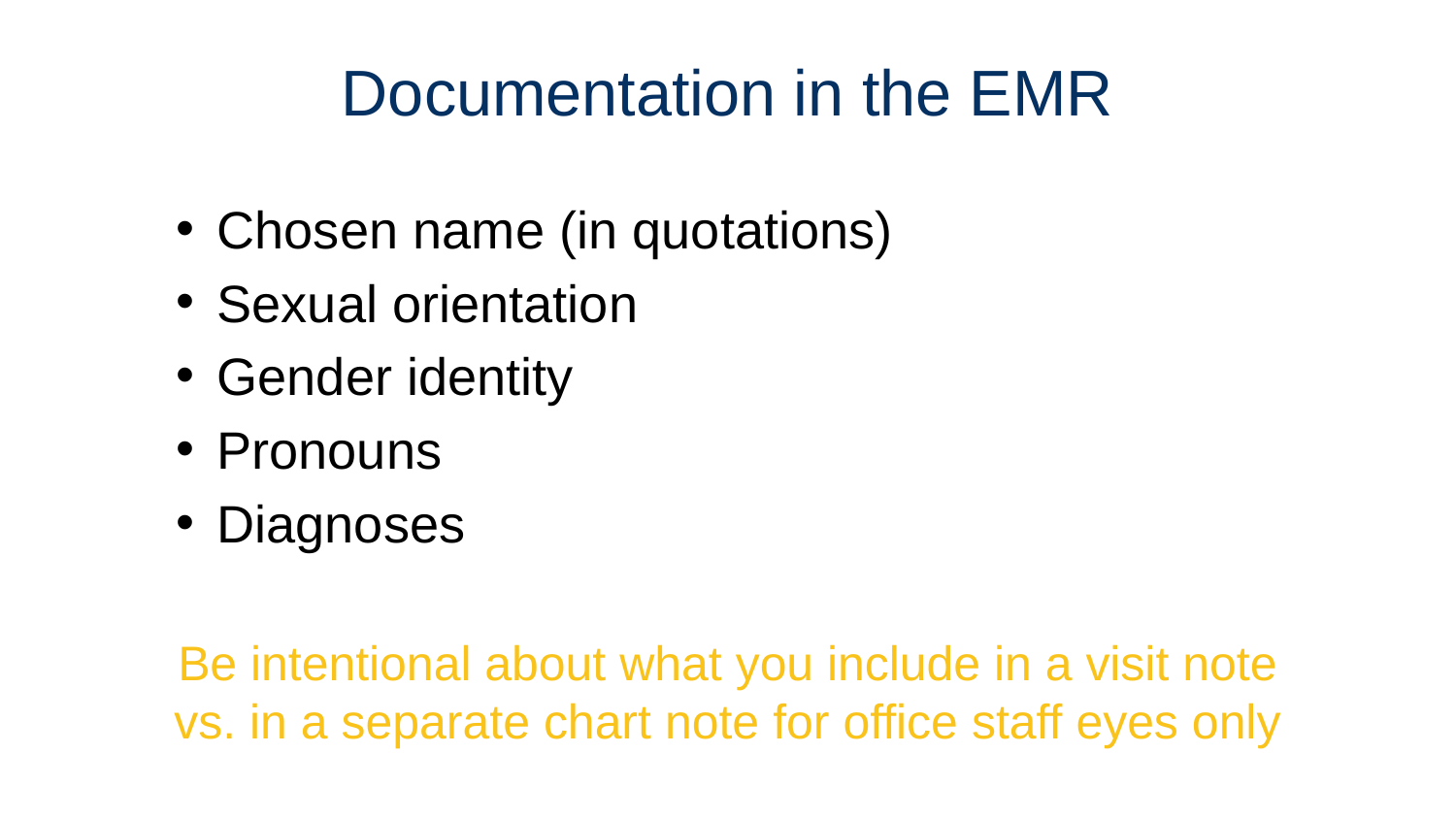

# Documentation in the EMR
Chosen name (in quotations)
Sexual orientation
Gender identity
Pronouns
Diagnoses
Be intentional about what you include in a visit note vs. in a separate chart note for office staff eyes only

## Slide 27
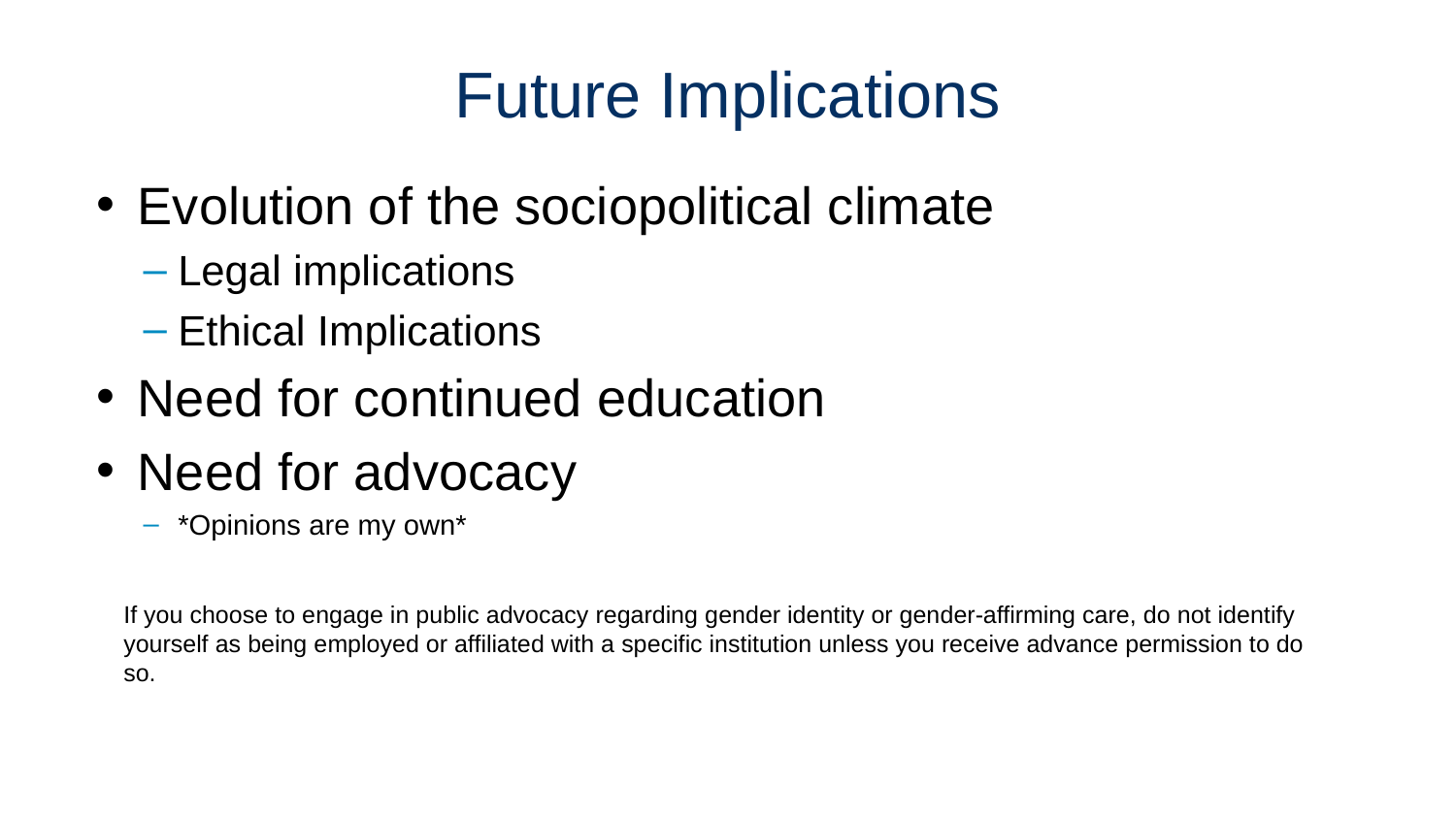

# Future Implications
Evolution of the sociopolitical climate
Legal implications
Ethical Implications
Need for continued education
Need for advocacy
*Opinions are my own*
If you choose to engage in public advocacy regarding gender identity or gender-affirming care, do not identify yourself as being employed or affiliated with a specific institution unless you receive advance permission to do so.

## Slide 28
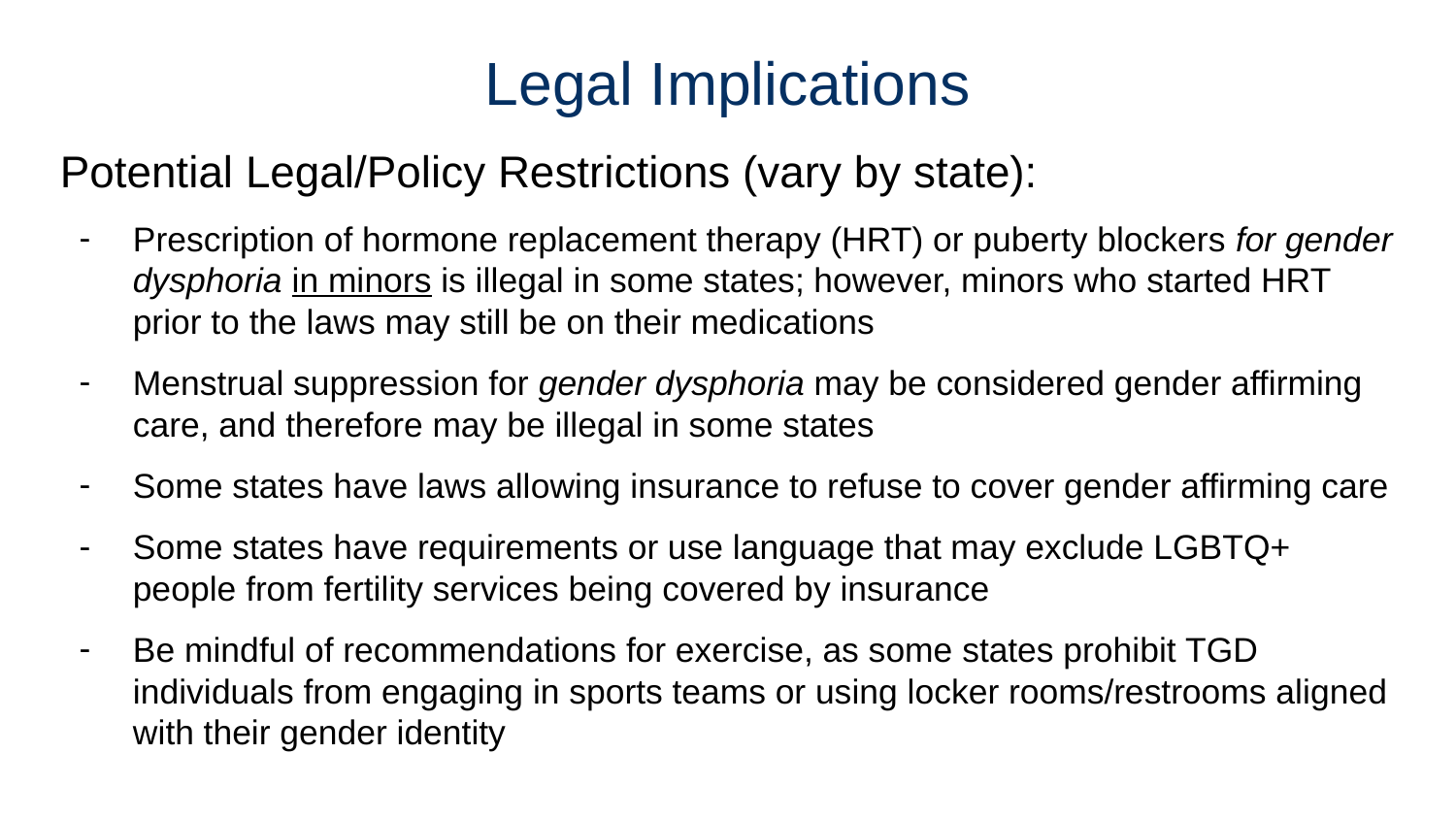

# Legal Implications
Potential Legal/Policy Restrictions (vary by state):
Prescription of hormone replacement therapy (HRT) or puberty blockers for gender dysphoria in minors is illegal in some states; however, minors who started HRT prior to the laws may still be on their medications
Menstrual suppression for gender dysphoria may be considered gender affirming care, and therefore may be illegal in some states
Some states have laws allowing insurance to refuse to cover gender affirming care
Some states have requirements or use language that may exclude LGBTQ+ people from fertility services being covered by insurance
Be mindful of recommendations for exercise, as some states prohibit TGD individuals from engaging in sports teams or using locker rooms/restrooms aligned with their gender identity

## Slide 29
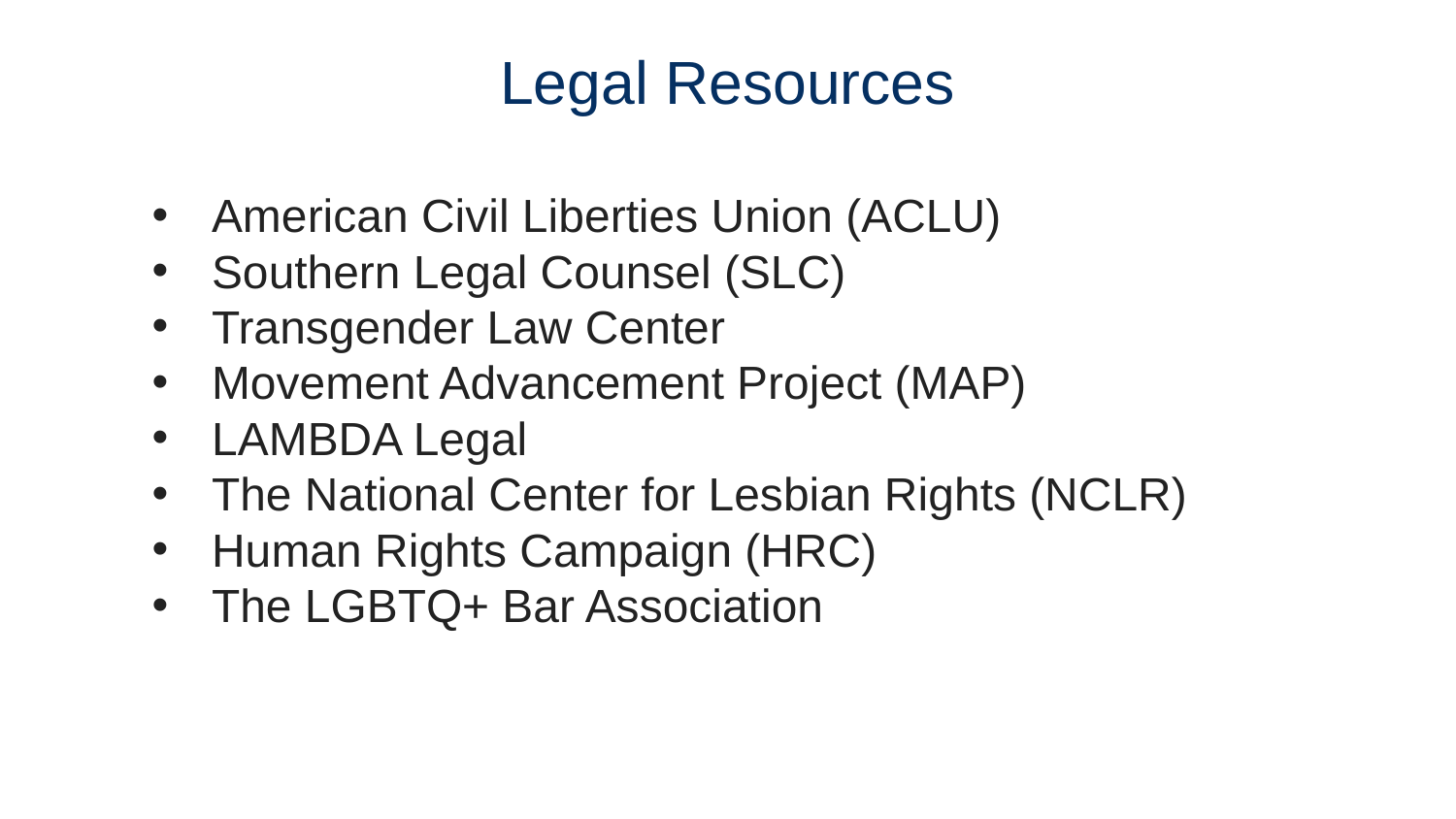

# Legal Resources
American Civil Liberties Union (ACLU)
Southern Legal Counsel (SLC)
Transgender Law Center
Movement Advancement Project (MAP)
LAMBDA Legal
The National Center for Lesbian Rights (NCLR)
Human Rights Campaign (HRC)
The LGBTQ+ Bar Association

## Slide 30
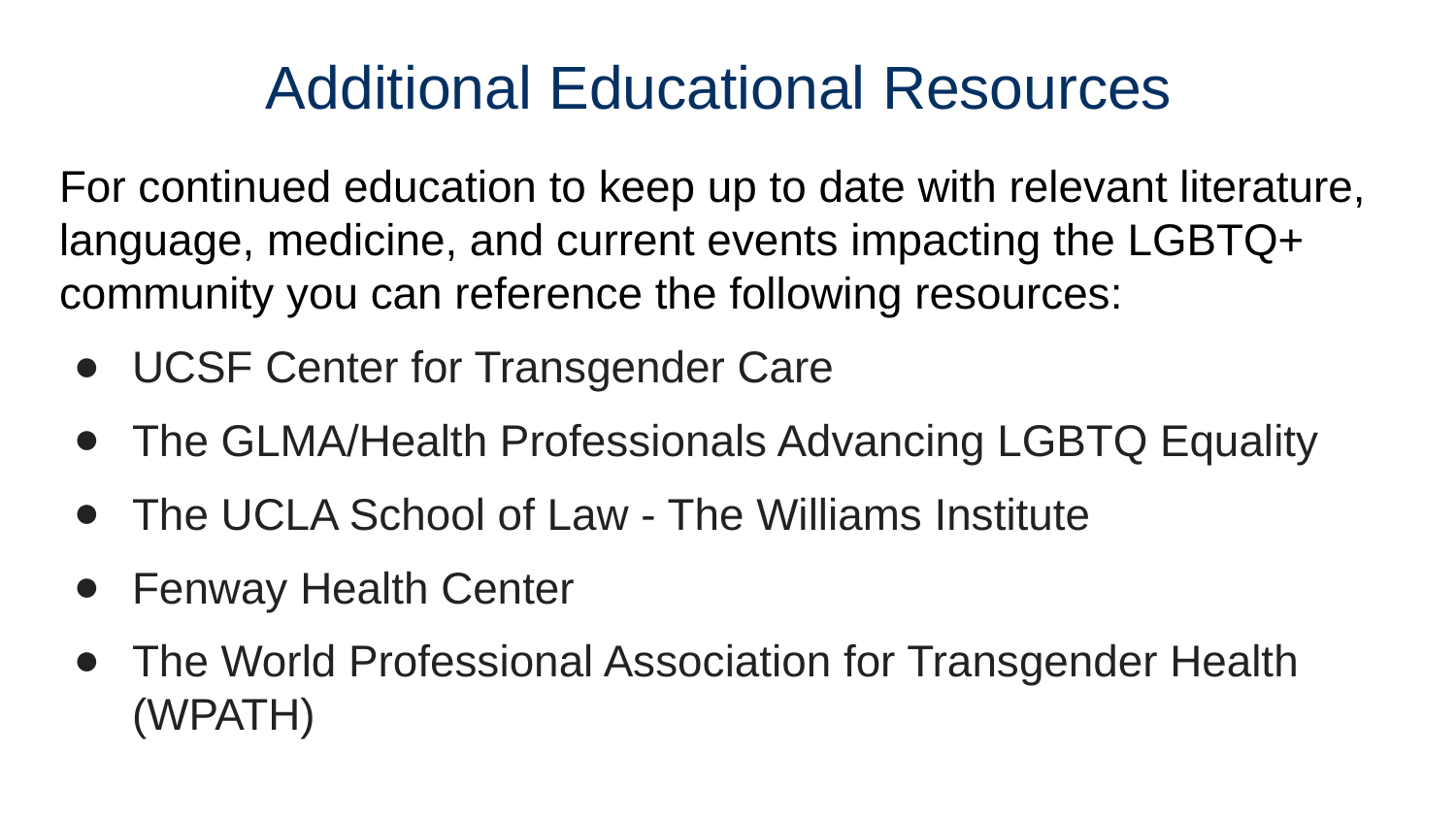

# Additional Educational Resources
For continued education to keep up to date with relevant literature, language, medicine, and current events impacting the LGBTQ+ community you can reference the following resources:
UCSF Center for Transgender Care
The GLMA/Health Professionals Advancing LGBTQ Equality
The UCLA School of Law - The Williams Institute
Fenway Health Center
The World Professional Association for Transgender Health (WPATH)
