# Supplementary material for: Simulation Bridges LGBTQ+ Educational Gaps in Gynecologic Care: Menstrual Suppression for a Gender and Sexually Diverse Patient
Source: MedEdPORTAL. 2025 Apr 1;21:11511. doi: 10.15766/mep_2374-8265.11511 (PMC11958776; doi:10.15766/mep_2374-8265.11511)
Supplement: Supplementary file 1 — SP Recruitment Materials and Guide.docxLGBTQ+ Resident Training Lecture.pptxResident Door Entry Instructions.docxSP Case.docxChecklist for Observers.docxExample Phrases.docxScripted Debrief.docxPre- and Postsurveys.docx [file mep_2374-8265.11511-s001.zip › C. Resident Door Entry Instructions.docx]

**Pre-briefing to be read to learners prior to the start of the scenario:**

Alexis “Alex” Smith is a 25-year-old G0 who was assigned female at birth (AFAB). The patient requests to be called Alex. Alex is a new patient to the practice who presents to discuss menstrual suppression. The goal of this scenario is to practice using inclusive language when speaking to gender diverse patients, assess goals of reproductive care with regards to menses, contraception and childbearing, use shared decision making to create a plan that aligns with patient goals and foster a welcoming environment. Gender diverse individuals often experience inequities and discrimination within the healthcare system. This simulation is designed to expose resident physicians to an authentic scenario that incorporates knowledge of gender diverse health issues along with providing residents an opportunity to enhance interpersonal and communication skills with patients who identify as gender diverse.

In this scenario, you will encounter things that may be artificial due to the use of standardized patients. Do your best to suspend reality and treat this scenario as real. While there is artifact, we aim to make the scene as real as possible. If at any point this evokes difficult memories of real-life situations and you are feeling overwhelmed, you may exit the scenario with no penalty. We believe all learners are capable individuals who try their best every day. Simulation is an excellent time to make mistakes and learn from them so that we may improve clinical care. All aspects of this simulation are only to be discussed within the simulation group and not anywhere else.

When you are ready, you may knock and enter.
