# Supplementary material for: Simulation Bridges LGBTQ+ Educational Gaps in Gynecologic Care: Menstrual Suppression for a Gender and Sexually Diverse Patient
Source: MedEdPORTAL. 2025 Apr 1;21:11511. doi: 10.15766/mep_2374-8265.11511 (PMC11958776; doi:10.15766/mep_2374-8265.11511)
Supplement: Supplementary file 1 — SP Recruitment Materials and Guide.docxLGBTQ+ Resident Training Lecture.pptxResident Door Entry Instructions.docxSP Case.docxChecklist for Observers.docxExample Phrases.docxScripted Debrief.docxPre- and Postsurveys.docx [file mep_2374-8265.11511-s001.zip › D. SP Case.docx]

Appendix D: Standardized Patient Case

Date: 05/05/2023

Primary Case Author: Tess Chase, MD

Secondary Case Author: Callie King, PhD

Standardized Patient Educator: Tess Chase, MD

Name of Case: LGBTQ+ Patients in Gynecologic Care: Menstrual Suppression for a Gender and Sexually Diverse Patient

Name of Educational and/or Assessment Activity: Simulation Supplements LGBTQ+ Gynecologic Care

Patient Name:

Legal name: Camielle Alexis Smith

Preferred name: Alex Smith

Chief Complaint: Desires menstrual suppression

Most Likely Diagnosis and Differential With Rationale From History and/or Physical Exam: This patient is experiencing gender dysphoria from their menses. They identify as non-binary and experience a depressed mood surrounding their menses and it is interfering with their quality of life.

Challenge Questions:

1. "Do I have to have a pelvic exam? I don't want one." If the learner says yes, then the standardized patient will ask "Why?"

2. "Would you be willing to prescribe my hormones?"

3. "Can I keep getting my care with you? If the learner says no, then the standardized patient will ask "Why?"

4. "My family is supportive but do you have any advice for coming out to my friends and romantic partners?"

Domains:

- Professionalism
- Communication and Interpersonal Skills
- Medical History
- Physical Exam
- Shared Decision-Making
- Patient Education
- Clinical Reasoning
- Documentation
- Handoff
- Presentation
- Other:

Type and Level of Learner: This case is appropriate for learners of all levels and is specifically written for those who will be providing gynecologic care such as OBGYN, family medicine and pediatrics residents.

Case Objectives:

a. Demonstrate the use of appropriate language in communicating with sexual and gender diverse patients (e.g., pronouns, chosen names, sexual terminology, etc.)
b. Utilize other communication skills and best practices that foster inclusivity and affirmation with sexual and gender diverse patients (e.g., gender neutral language, trauma-informed care, appropriate and affirming language for sexual history collection)
c. Engage in appropriate education and shared decision-making regarding contraceptive options in transgender and gender diverse patients assigned female at birth desiring amenorrhea

d. Use clinical reasoning and shared decision-making to develop a treatment plan specific to the patient’s needs.

| SETTING: outpatient, in patient, ED, home, nursing home, rehab, group, etc. | Office in a typical outpatient exam room |
| --- | --- |
| PATIENT PROFILE: | |
| Age range | 20-30 |
| Religious/spiritual background | all may be used |
| Sex (e.g., male, female, intersex, transwoman, transman) | Assigned female at birth |
| Sexual orientation (e.g., heterosexual, lesbian, gay, bisexual, pansexual, queer, asexual) | Pansexual |
| Gender expression (e.g., man, woman, genderqueer) | Non-binary |
| Race and ethnicity (e.g., to promote educational diversity, we use a diverse pool of SPs.) | all may be used |
| Physical description (e.g., BMI, height range) | all may be used |
| Physical limitations | all may be used |
| Patient appearance (e.g., disheveled, hospital gown, business casual, casual) | Androgynous (not too feminine or masculine), loose-fitting clothing |
| Moulage + location (e.g., none, bruises, scars, body piercing, tattoos) | all may be used |
| Affect (e.g., pleasant, cooperative) | Nervous but cooperative |
| Family group (e.g., who is family, who they live with) | all may be used |
| Education | Working on a Master’s degree in education |
| Level of health literacy | Average |
| Employment, if any - present and past, noting any current stresses | Works at a Bookstore |
| Home/homeless - type of dwelling, number of stories, owned or rented | Lives in an apartment |
| Financial situation - any current stresses | Lives within their means, no major financial stressors |
| Insurance status (e.g., un/under/insured, public/private, HMO/PPO) | Private insurance through work |
| Habits (i.e., diet, exercise, caffeine, smoking, alcohol, drugs) | Drinks caffeinated tea while at work. Does not smoke, drink alcohol, or do drugs. |
| Activities (i.e., hobbies, sports, clubs, friends) | Enjoys reading and crocheting |
| Typical day - what is the usual daily routine | The patient attends classes on Monday/Wednesday/Friday and works at the bookstore on Tuesday/Thursday/Saturday. |

| CASE INFORMATION | |
| --- | --- |
| Chief Concern: What the patient will say when greeted by the student. The patient’s primary reason for seeking medical care often stated in their own words. | “Hello, I am here because I feel really awful when I get my period and in the week before my period. I want my periods to stop. They make me feel really bad about myself and cause significant gender dysphoria.” |
| Additional Concerns: Other, if any, concerns the patient has today (i.e., symptoms, requests, expectations, etc.) that will become part of set agenda. | They are concerned about how their period affects their mood. They have a lot of anxiety in the days preceding their period and feel down once it comes. They have had to call out of work a few times and this is concerning to them because they worry it will affect their upcoming performance review. They are up for promotion to Bookstore Manager.  They have never had a pap smear before and are nervous about having an exam performed. They do not want an examination today and will request to come back for screening at another time if an exam is advised. They are unwilling to have a gynecologic examination today.  *They hope that there is a medication they can take to stop periods.*  *They are feeling nervous and out of place at the GYN office. They will not divulge a lot of information unless directly asked or the provider makes you feel welcome and comfortable.*  *If the provider is empathetic, welcoming and uses inclusive language, then you will relax during the scenario and open up/offer more information. SP will express relief when the provider expresses empathy and uses gender inclusive language. If provider asks about pronouns, uses inclusive language, makes SP feel welcome, then SP may open up/appear more comfortable.*  *Information:*  *Bring up only if asked: That they are interested in men or women for romantic relationships and that they may enter into a romantic relationship soon.*  *Give spontaneously:  Periods give them a lot of anxiety and depression. They experience dysphoria with menses. After discussing menstrual suppression, they can ask about any other health maintenance requirements if pap smear screening is not brought up.* |
| THE PATIENT’S STORY: | Well, this has been going on for a while. I have never really felt right in my body since I was a young child. I can't say the exact age. I just felt like the body parts did not fit until I learned about gender identity. I identify as non-binary and I have been using testosterone to masculinize my facial features and voice. I thought it would take my period away but when it did not, all the feelings of anxiety, depression, and dysphoria came back.  Typically, I start to feel more dysphoric the week before my period and when I am on my period. I get bad cramps that improve with ibuprofen. I work at a bookstore and when I am on my period I don't even want to go to work because I am so upset about it. I can't keep calling out for a week every month or else I am going to lose my job and I won't be able to finish school. I really hope you can give me something to take my periods away. I hate them. It's really affecting my quality of life.  My family knows about my gender identity, and they are supportive. It was actually my mom who recommended I come to the gynecologist. I really do not want to be here, but she said it could help. |
| HISTORY OF PRESENT ILLNESS:  A 25-year-old patient presents to discuss menstrual suppression. This is their first visit to the GYN. Their periods occur every 28 days and last 5 days in duration. They notice that their mood is particularly bad during the few days before and while on their period.  Their periods are also painful and respond well to ibuprofen. They have never had a pap smear before and are nervous about having an exam performed. They are interested in sexual relationships with both men and women. | |
| Onset (when; gradual or sudden) | The symptoms have been going on since menarche. |
| Setting | Symptoms are present regardless of the setting. |
| Duration (how long) | The mood symptoms start 1 week prior to menses and while on menses. The cramping is only present during menses. They have felt that they do not identify with their assigned sex at birth since they were a young child. |
| Time relationships (frequency, constant or intermittent) | The mood symptoms wax and wane but are worse when they are alone or not distracted by work. |
| Location | Cramping is in the middle of the lower abdomen. |
| Radiation | None |
| Quality | Crampy pain |
| Amount | 6/10 |
| Aggravated by what | Nothing |
| Relieved by what | Ibuprofen helps some |
| Associated with what | Menses |
| Attitude | They feel very uncomfortable having a period and it is contributing to feelings of anxiety, depression and dysphoria. |
| Overall course | Symptoms occur each month prior to and during menses. |
| REVIEW OF SYSTEMS: | |
|  | Negative for nausea, vomiting, constipation, diarrhea, dysuria or hematuria.  Positive for pelvic pain with menses |
|  | Positive for anxiety |
| Past medical history: | Anxiety, depression |
| Medication allergies (name and reaction): | None |
| Environmental allergies (name and reaction): | None |
| Illnesses: | None |
| Vaccinations: | Unknown |
| Surgeries: | None |
| Accidents/injuries/trauma: | None |
| Hospitalization: | None |
| Inclusive sexual and reproductive history | |
| Sexual practices  Sexual partners  Protection: Use of safer sex practices  Use of birth control if appropriate  Risk of intimate partner violence | They have sex with men and women.  They have had 3 sexual partners and are considering entering a new romantic relationship with an assigned male at birth (AMAB) partner. They do not use condoms and are not on any other form of birth control. They have healthy relationships at home and denied safety concerns with this new partner. |
| OB/GYN history | Age of onset of menses: 13  Age of menopause: n/a  Number of pregnancies: 0  Number of live births: n/a  Number of miscarriages: n/a  Number of abortions: n/a |
| Medications | Prescription/dose/reason- none  Over the counter/dose/reason – n/a  Herbs/supplements/dose/reason  Other: Testosterone Injections for gender affirming hormone treatment |
| Immunizations | - Tetanus - Flu - Hepatitis - Pneumovax - HPV - Other: Unknown |
| Tobacco products:   - Cigarettes - Cigar - Pipe - Chew - E-cigarettes | - Never - Past - year started/year quit - Current   - Quantity   - # of years |
| Alcohol   - Beer - Wine - Liquor - Other | - Never - Past - year started/year quit - Current   - Quantity   - # of years |
| Drugs   - Weed - Cocaine - Heroin - Meth - IV - Inhalants - Other | - Never - Past - year started/year quit - Current   - Quantity   - # of years |
| Diet (describe) | Omnivore diet eats fast food 1x per week. |
| Exercise (describe) | None |
| List any other important social history or information important to this case | They are interested in romantic and sexual relationships with men and women. They are not currently partnered but have recently met someone new and are interested in pursuing that relationship. Lives alone in an apartment with their cat.  Parents are supportive of their gender identity. They have 2 siblings who are also supportive. |
| Family history | Noncontributory |
| Mother, father, siblings, grandparents, and other significant findings | n/a |
| Physical Exam – Findings are known as no physical exam will occur during simulation.  General: NAD  HEENT: Normocephalic, atraumatic  Neck: Normal  Lungs: CTABL, no acute distress, no increased work of breathing  Cardiovascular: RRR, no M/G/R  Abdomen: Soft, non tender, non distended, no masses, rebound or guarding  Neurological: CN 2-12 grossly intact  Skin; Normal  GU : N/A, no exam to be performed  Psychiatric :  Mood: Anxious and hesitant.  Attitude: Hopeful that medical therapy may work for you.  Behavior: Cooperative | |
| PHYSICAL EXAM FINDINGS |  |
| 1. Written in layperson’s terms | The physician examination is normal for the nerves, heart, lungs and abdomen. |
| 1. General appearance - affect, appearance, position of patient at opening (i.e., sitting, lying down, holding abdomen, etc.) | Sitting on the exam table |
| 1. Vital signs | Temp 98.0, HR 95, BP 121/78 |
| 1. Specific findings and affect | Appears anxious and reserved |
| 1. Response to certain physical movements | None |
| CRITICAL ACTION CHECKLIST | Learners introduce themselves, confirm patient’s preferred name and pronouns. These should be used throughout the visit.  Learners will begin taking a history.  Standardized patient will give history and discuss concerns about menses and worry about being at the GYN.  Learners will use shared decision making to create a personalized plan of care with the patient  Patient will ask about examination/ health maintenance screening if not brought up by the learners.  The patient will decline pelvic examination if asked.  Scene will end after the plan is created and the patient is satisfied with the plan of care. |
| DIAGNOSIS AND DIFFERENTIAL |  |
| Diagnosis with support from positive and negative history and PE findings | Potential Diagnoses: Gender dysphoria, premenstrual dysphoric disorder, anxiety, depression, dysmenorrhea, endometriosis, constipation |
| Differential with support from positive and negative history and PE findings | Gender dysphoria: This is the diagnosis based on patient’s history and normal examination. History is the most important factor in making this diagnosis. The patient has felt that they do not identify with their assigned sex at birth since early childhood AND it causes significant distress.  PMDD: While the menses interfere with the mood, the patient can also identify gender dysphoria as the primary driving factor rather than the menstrual cycle itself.  Anxiety and Depression: May also be a contributor, however, the patient notes it worsens with the menstrual cycle.  Dysmenorrhea: Likely based on the mild pain symptoms which only occur on menses and improve with ibuprofen  Endometriosis: Less likely given that the pain does not occur outside of menses and there are no other associated symptoms such as nausea, vomiting, constipation or diarrhea. They have also not tried any menstrual suppression to see if the pain improves. |
| MANAGEMENT OR DIAGNOSTIC PLAN | Develop a care plan with the patient with review of all forms of hormonal menstrual suppression. Use shared decision making to choose (likely) a progesterone only form of contraception which will also be used for menstrual suppression. The scenario may end successfully with the choice of norethindrone .35mcg, Depo Provera, Nexplanon or progesterone IUD. |
| PROFESSIONALISM ISSUES OR CHALLENGES | If the learner misgenders the patient repeatedly or does not make the SP feel welcome, the SP will shut down. The more respectful language that is used will develop a good rapport with the patient for a successful visit. |
