# Supplementary material for: Simulation Bridges LGBTQ+ Educational Gaps in Gynecologic Care: Menstrual Suppression for a Gender and Sexually Diverse Patient
Source: MedEdPORTAL. 2025 Apr 1;21:11511. doi: 10.15766/mep_2374-8265.11511 (PMC11958776; doi:10.15766/mep_2374-8265.11511)
Supplement: Supplementary file 1 — SP Recruitment Materials and Guide.docxLGBTQ+ Resident Training Lecture.pptxResident Door Entry Instructions.docxSP Case.docxChecklist for Observers.docxExample Phrases.docxScripted Debrief.docxPre- and Postsurveys.docx [file mep_2374-8265.11511-s001.zip › E. Checklist for Observers.docx]

Appendix E: Checklist for Observers

As you observe the simulation, please reflect on strengths and areas for growth of your cohorts and be prepared to share during the debriefing. Reference the below list of items that should be included in the encounter:

Learning objectives:

- Demonstrate the use of appropriate language in communicating with sexual and gender diverse patients (e.g., pronouns, chosen names, sexual terminology, etc.)
- Utilize other communication skills and best practices that foster inclusivity and affirmation with sexual and gender diverse patients (e.g., gender neutral language, trauma-informed care, appropriate and affirming language for sexual history collection)
- Engage in appropriate education and shared decision-making regarding contraceptive options in transgender and gender diverse patients assigned female at birth desiring amenorrhea.

Skills:

- Introduces self with name and pronouns
- Inquires about patient’s chosen name and pronouns using affirming language
- Inquires about desire for name and pronouns to be included in patient medical record
- Inquires about patient’s reason for seeking medical care
- Uses inclusive language when building rapport with patient and gathering history
- Uses warm and welcoming body language to promote relaxation and build trust
- Uses gender neutral and/or anatomically correct language when referring to medical concerns and body parts
- Gathers specific sexual history (5 P’s: partners, practices, protection from STIs, past history of STIs, and pregnancy intention) and provides appropriate resources and psychoeducation as necessary
- Assesses for mood disturbances surrounding the menstrual cycle
- Inquires about past, present, and intent for future use of HRT (e.g., testosterone)
- Provides patient education about the impact of HRT on the menstrual cycle, if applicable
- Provides appropriate options for menstrual suppression in line with patient’s goals
- Adequately describes when physical exams are necessary and collaboratively decides if an exam is necessary in today’s visit
- Provides opportunity to discuss patient concerns or discomfort with physical exam (or any part of the visit) and addresses concerns with empathy
