# Supplementary material for: Simulation Bridges LGBTQ+ Educational Gaps in Gynecologic Care: Menstrual Suppression for a Gender and Sexually Diverse Patient
Source: MedEdPORTAL. 2025 Apr 1;21:11511. doi: 10.15766/mep_2374-8265.11511 (PMC11958776; doi:10.15766/mep_2374-8265.11511)
Supplement: Supplementary file 1 — SP Recruitment Materials and Guide.docxLGBTQ+ Resident Training Lecture.pptxResident Door Entry Instructions.docxSP Case.docxChecklist for Observers.docxExample Phrases.docxScripted Debrief.docxPre- and Postsurveys.docx [file mep_2374-8265.11511-s001.zip › F. Example Phrases.docx]

**Appendix F: Examples of phrases to use with Transgender Patients**

**Introductions**

“Hello! My name is Dr. __ and I use ___/___ pronouns. I see your legal name in your chart, but what would you like me to call you?”

IF different than legal name, ask – “Great, would you like me to document that in your medical record so it’s in your chart for other providers in our system to see?”

Always check paperwork for pronouns first! If you need to ask, say “What are your pronouns?” or “What pronouns do you use?” instead of “What are your preferred pronouns?”

- "Welcome! We are happy to have you here! I know it can be really intimidating to be here in a traditionally female space. We will just be having a conversation today, no examination. I typically only need to do an exam if..."

**Information and Rapport Building**

- "Anyone who has a cervix needs a pap smear starting at age 21"

- "Periods are the actual worst!"

- "This is a safe place for you to come if you ever need help or you do not feel comfortable talking to (parents, etc). I am a trusted adult and have your best interest at heart"

- “Before we begin, it’s helpful for me to know if you experience any kind of dysphoria or discomfort when talking about your body. Can you tell me if there are specific words or areas of your body that might be more triggering you’d like me to avoid?”

o “Great, thank you for letting me know. We shouldn’t have to focus on that today.” OR

o “During the exam, I will need to ____ (examine, touch, discuss… etc.) that area due to the nature of this visit. How can I make this more comfortable for you? Is there another word you’d like me to use to refer to that area?”

**History Collection**

- Menstrual History:

“How often do you bleed?”

“Have you ever utilized anything to stop you from bleeding, like birth control or implants?”

- Sexual History:

“Are you sexually active?”

IF yes: “Do you have sex with people with a penis, with a vagina, or both?”

“During sex, do any parts of your body enter your partners body (or vice versa), such as their genitals, anus, or mouth?”

“What kinds of safe sex practices do you use? (ex., condoms, dental dams, etc.)”

“If you share any kind of sexual pleasure device (or toy) during sex or between sexual partners, are you using protection on those as well?”

“What kinds of contraception do you use?”

“When was your last screening for STIs and/or HIV?”

“Do you have a history of sexual trauma?”

“You do not need to tell me about it, it’s just helpful for me to know if that is part of your history so I can provide the best quality of care.”

Sexual Experiences

“Do you typically feel pleasure when having sex?”

“Do you experience any pain or discomfort when having sex?”

Fertility Discussions

"Would you ever want to be pregnant?"

“Are you interested in learning about fertility options or family planning?”

Hormone Therapy

“Have you ever utilized hormone therapy for any reason?”

IF yes: “For how long?” “Why/When did you stop?” “What was your experience like?”

"Do you have any plans for testosterone therapy?"

**Physical Examination**

Key component: Narrate your actions before you do them and respect the language/boundaries they asked of you as is feasible.

“It’s time for the physical exam. I am going to ___ (describe what you will do using anatomical or neutral language). The reason I have to do this is because ____/I’m just checking it out to make sure everything feels like it should.”

“What questions do you have about this before we get started? Is there anything I can do that would make this more comfortable for you?”

Try to avoid Yes/No (close-ended) questions. Patients are more likely to say no to these and not ask what they truly want to.

Instead of “do you have any questions?” ask: “what questions do you have?”

- Language examples:

“inside of you” “in your first hole” instead of “in your vagina”

“chest” instead of “breast”
